# Next generation approaches in cancer immunotherapy targeting mechanisms beyond PD1 and PDL1

**DOI:** 10.1007/s12672-026-04852-1

**Published:** 2026-03-14

**Authors:** Mohamed El-Tanani, Shakta Mani Satyam, Syed Arman Rabbani, Imran Rashid Rangraze, Ismail Ibrahim Ali Matalka, Frezah Muhana, Yahia El-Tanani, Alaa A. A. Aljabali, Mohammad Ahmed Khan, Suhel Parvez, Thantrira Porntaveetus

**Affiliations:** 1 RAK College of Pharmacy, RAK Medical and Health Sciences University, Ras Al Khaimah, 11172 United Arab Emirates; 2 Department of Pharmacology, Translational Medical Research Centre & Central Animal Research Facility, RAK College of Medical Sciences, RAK Medical and Health Sciences University, Ras Al Khaimah, 11172 United Arab Emirates; 3 Department of Clinical Pharmacy, RAK College of Pharmacy, Ras Al Khaimah Medical and Health Sciences University, Ras Al Khaimah, United Arab Emirates; 4 Department of Internal Medicine, RAK College of Medical Sciences, RAK Medical and Health Sciences University, Ras Al Khaimah, United Arab Emirates; 5 Department of Pathology, RAK College of Medical Sciences, RAK Medical and Health Sciences University, Ras Al Khaimah, United Arab Emirates; 6 Princess Sarvath Community College, Amman, Jordan; 7 Royal Cornwall Hospital Trust, NHS, Truro, UK; 8 Department of Pharmaceutics and Pharmaceutical Technology, Faculty of Pharmacy, Yarmouk University, Irbid, Jordan; 9 School of Pharmaceutical Education and Research, Jamia Hamdard, New Delhi, India; 10 School of Chemical and Life Sciences, Jamia Hamdard, New Delhi, India; 11 Center of Excellence in Precision Medicine and Digital Health, Department of Physiology, Geriatric Dentistry and Special Patients Care Program, Faculty of Dentistry, Chulalongkorn University, Bangkok, Thailand

**Keywords:** Cancer immunotherapy, Immune checkpoint inhibitors, Tumor microenvironment, Combination therapy, Precision oncology, Artificial intelligence in oncology

## Abstract

Cancer immunotherapy has revolutionized oncology by leveraging the immune system’s ability to recognize and eliminate tumor cells. Among the most impactful advances are immune checkpoint inhibitors that target the programmed death-1 (PD-1) receptor and its ligand PD-L1, which can restore T cell activity and generate durable responses across several cancer types. However, their effectiveness is limited by both primary and acquired resistance, restricting long-term benefit to a subset of patients. This review highlights current progress in cancer immunotherapy and explores emerging strategies beyond PD-1/PD-L1 blockade. Novel inhibitory and co-stimulatory immune checkpoints such as lymphocyte activation gene-3 (LAG-3), T cell immunoglobulin mucin-3 (TIM-3), T cell immunoreceptor with Ig and ITIM domains (TIGIT), OX40, CD137 (4-1BB), and CD40 are examined for their potential to enhance anti-tumor immunity. The rationale for combining immunotherapy with chemotherapy, radiotherapy, targeted therapies, oncolytic viruses, and neoantigen vaccines is discussed in the context of overcoming resistance. Additional emphasis is placed on modifying the tumor microenvironment, targeting tumor metabolism, and overcoming stromal barriers. Advanced delivery systems such as chimeric antigen receptor (CAR) T cells, bispecific antibodies, and nanoparticle-based platforms offer improved specificity and reduced toxicity. Biomarker-guided precision immuno-oncology using tumor mutational burden, microsatellite instability, and immune gene signatures is advancing clinical decision-making. Gut microbiota modulation and artificial intelligence-based tools are also emerging as critical components in optimizing therapeutic outcomes. Collectively, this review proposes a multidimensional and personalized immunotherapy paradigm aimed at broadening clinical efficacy and overcoming resistance beyond conventional PD-1/PD-L1 inhibition.

**Graphical Abstract:**

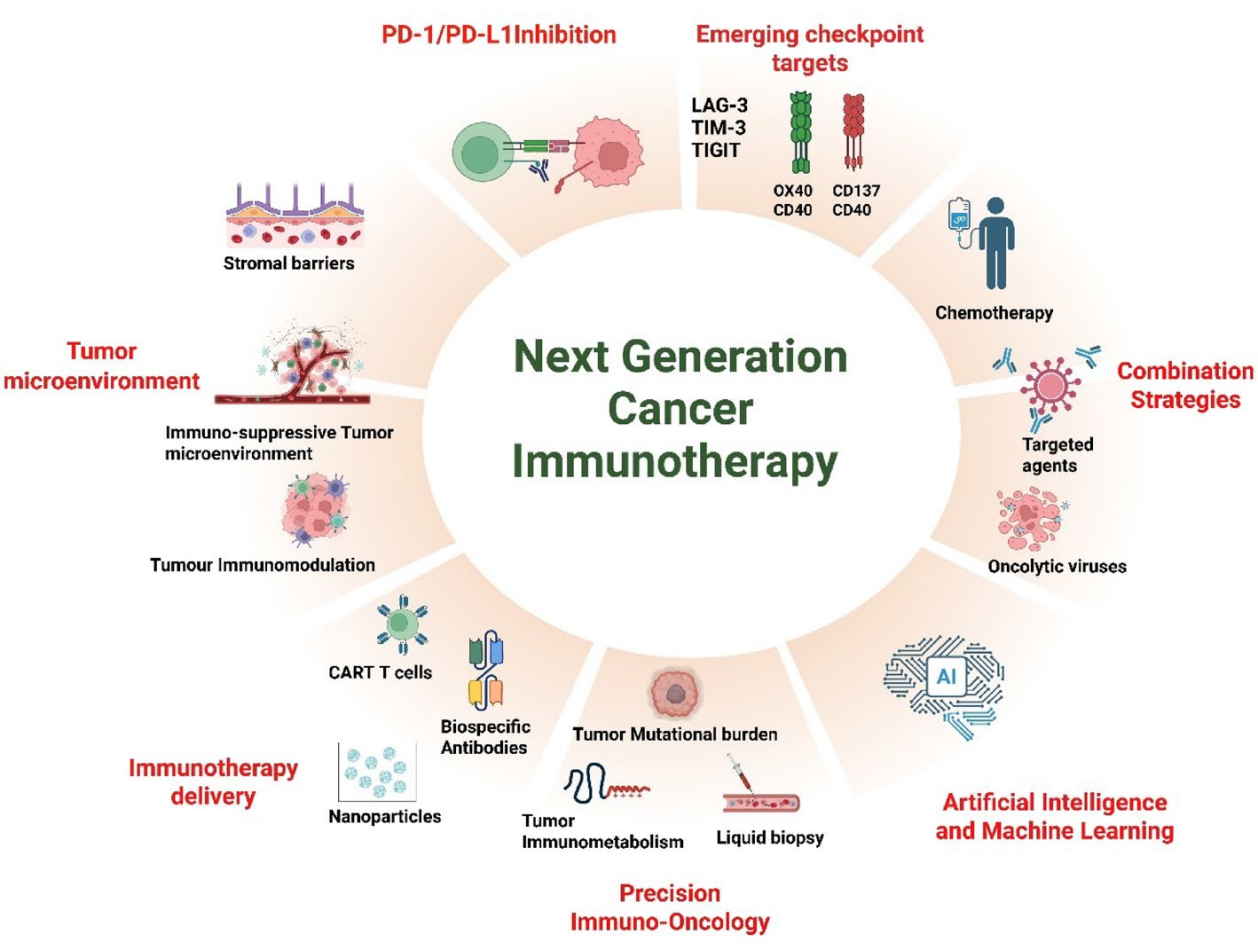

## Highlights

- Next-gen immune checkpoints target resistance beyond PD-1/PD-L1 axis.
- Immunometabolic rewiring emerges as a novel immunotherapy strategy.
- Precision combinations with oncolytic viruses and neoantigens show promise.
- AI-driven platforms optimize immunotherapy response and toxicity prediction.
- Gut microbiome modulation reshapes host-immune-tumor interactions.

## Introduction

 The immune system serves as the body’s foremost defense against oncogenic transformation, engaging in a constant surveillance process termed cancer immunosurveillance. This intricate mechanism involves the identification and elimination of nascent tumor cells by immune effectors such as cytotoxic T lymphocytes, natural killer (NK) cells, macrophages, and dendritic cells [1]. T cells recognize tumor-associated antigens (TAAs) presented via major histocompatibility complex (MHC) molecules and induce targeted cytolysis of transformed cells [2, 3]. NK cells, in contrast, act as sentinels of the innate immune system capable of destroying malignant cells that downregulate MHC expression—a common mechanism of immune escape [4]. However, as cancer evolves, tumor cells acquire adaptive mechanisms that subvert immune recognition. These include loss of antigenicity, downregulation of antigen-presenting machinery, secretion of immunosuppressive cytokines (such as TGF-β and IL-10), and recruitment of regulatory immune subsets including Tregs and myeloid-derived suppressor cells (MDSCs) [5, 6]. This complex equilibrium between tumor elimination and immune evasion forms the basis of cancer immunoediting, which encompasses the phases of elimination, equilibrium, and escape [7–9]. The understanding of this dynamic process underscores the imperative for immunotherapeutic interventions that not only restore but also amplify anti-tumor immune competence.

**Fig. 1:**
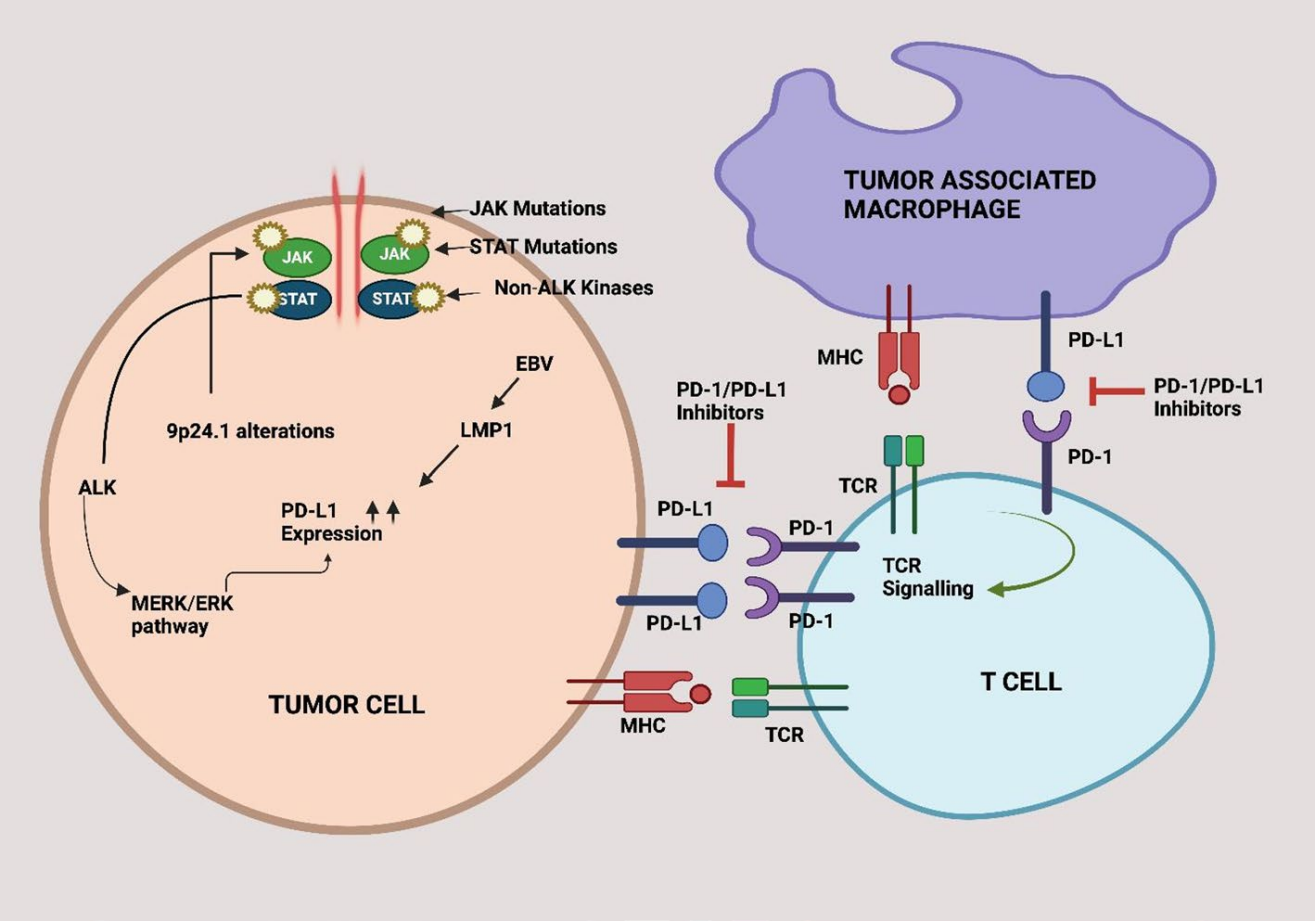
Mechanisms underlying PD-L1 upregulation and immune evasion in cancer

In recent decades, immunotherapy has emerged as a transformative pillar in oncology, fundamentally reshaping the clinical management of multiple malignancies. Unlike cytotoxic chemotherapy and radiation, which act directly on tumor cells but often compromise normal tissues, immunotherapies leverage the host’s immune system to achieve selective tumor targeting and durable disease control. Through the induction of immunological memory, these approaches can yield long-lasting protection even after cessation of therapy, offering a paradigm shift from palliative to potentially curative interventions. Among the most clinically validated immunotherapies are immune checkpoint inhibitors (ICIs), which counteract inhibitory signaling pathways that restrain T-cell activity [10]. The Programmed Death-1 (PD-1)/Programmed Death Ligand-1 (PD-L1) axis represents a central immune checkpoint exploited by tumors to evade immune destruction. PD-1 is an inhibitory receptor expressed on activated T cells, B cells, and NK cells; engagement with its ligands PD-L1 or PD-L2—often upregulated on tumor cells or tumor-associated macrophages—results in attenuation of T-cell receptor (TCR) signaling, cytokine suppression, and induction of T-cell exhaustion (Fig. 1) [11, 12].

This schematic illustrates how tumor cells increase PD-L1 expression through pathways such as JAK/STAT, ALK signaling, and genetic alterations (e.g., 9p24.1). PD-L1 on tumor cells binds to PD-1 on T cells and tumor-associated macrophages, inhibiting TCR signaling and promoting T cell exhaustion. PD-1/PD-L1 inhibitors block this interaction, restoring anti-tumor immunity.

**Fig. 2:**
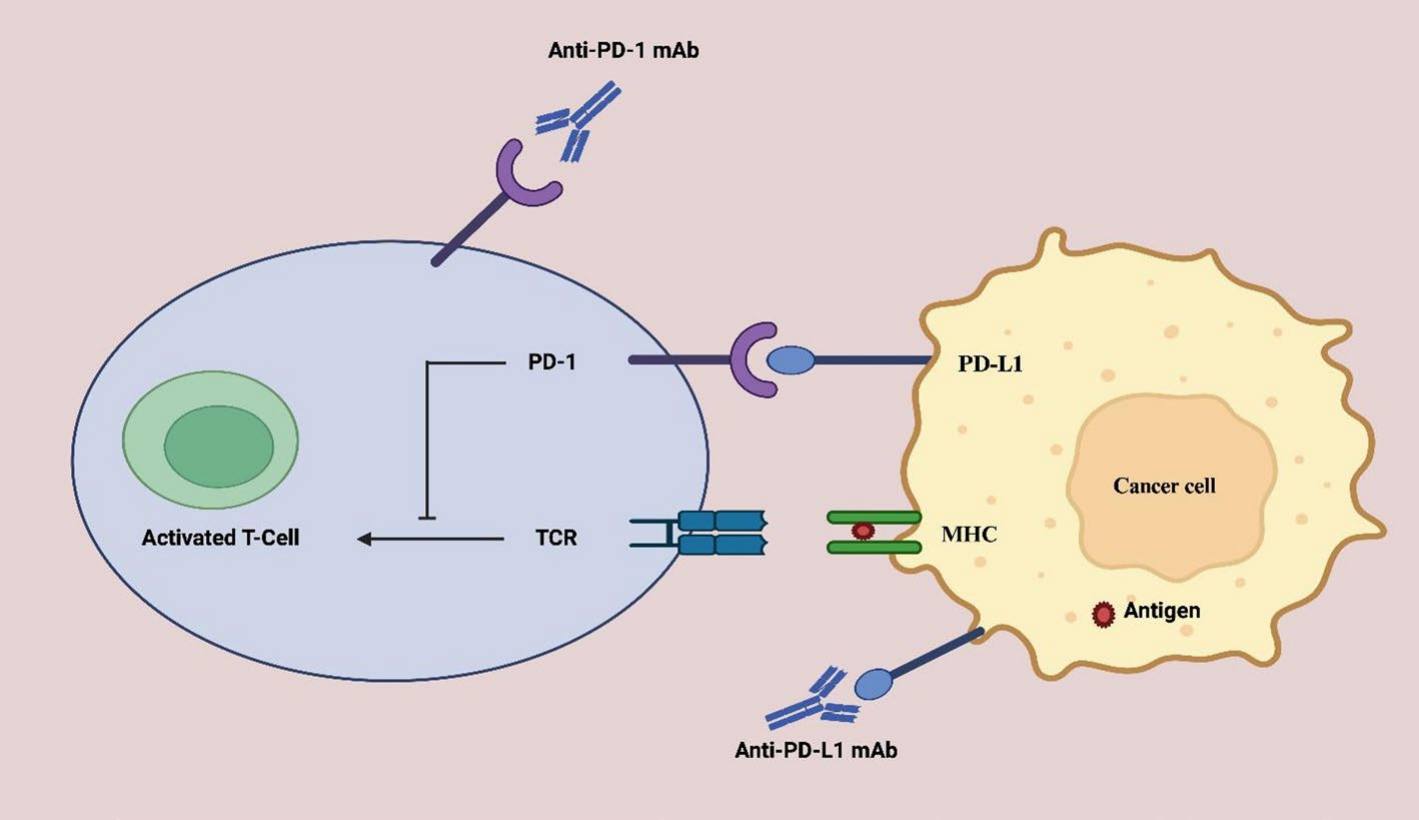
Mechanism of action of PD-1/PD-L1 inhibitors in T-cell-mediated tumor immunity

The clinical success of ICIs such as nivolumab and pembrolizumab has been remarkable, producing durable responses and survival benefits in malignancies including melanoma, non-small cell lung carcinoma (NSCLC), renal cell carcinoma, and Hodgkin lymphoma [10, 13]. Figure 2 conceptually depicts the mechanism of PD-1/PD-L1 blockade in T-cell-mediated tumor immunity, wherein antibodies disrupt inhibitory receptor–ligand engagement, enabling T-cell activation, proliferation, and tumor lysis. Beyond prolonging overall survival, ICIs have introduced the possibility of long-term remission, often outlasting treatment duration—a reflection of immune memory’s therapeutic potential [14].

Cancer cells express PD-L1 to engage PD-1 receptors on activated T cells, suppressing their cytotoxic activity. Anti-PD-1 and anti-PD-L1 monoclonal antibodies block this interaction, allowing TCR signaling and T cell-mediated tumor cell killing.

Nevertheless, despite these landmark advances, only a fraction of patients derive sustained benefit from checkpoint inhibition, and response rates remain heterogeneous across tumor types [15, 16]. Intrinsic and acquired resistance to ICIs arises from multifactorial determinants including defective antigen presentation (e.g., β2-microglobulin loss), mutations in interferon signaling pathways, an immunosuppressive tumor microenvironment (TME) enriched with Tregs and MDSCs, and metabolic competition within the TME that depletes key nutrients such as glucose and tryptophan essential for effector T-cell function [10, 17–19]. Furthermore, tumors characterized by a paucity of immune infiltration—often termed “immune-cold”—exhibit minimal responsiveness to checkpoint blockade [20, 21]. These observations underscore a critical unmet need to extend the benefits of immunotherapy beyond PD-1/PD-L1 pathways by exploring next-generation strategies that engage alternative immune mechanisms.

Recent studies have elucidated that immune resistance is not confined to checkpoint signaling but extends to deeper immunoregulatory networks involving myeloid checkpoints, co-stimulatory molecules, innate sensing pathways, and tumor metabolism [19, 22, 23]. For instance, the TIM-3 (T-cell immunoglobulin and mucin domain-3) and LAG-3 (lymphocyte activation gene-3) pathways have emerged as crucial mediators of T-cell exhaustion that synergize with PD-1 to sustain immune suppression [24–28]. Therapeutic targeting of LAG-3—through agents such as relatlimab—has demonstrated promising activity in combination with nivolumab, signifying the potential of dual-checkpoint inhibition to overcome adaptive resistance [29, 30]. Similarly, TIGIT blockade has gained traction for its ability to restore NK and T-cell cytotoxicity by disrupting inhibitory signaling through CD155/CD112 interactions [31–33].

Parallel to adaptive immune modulation, the reprogramming of innate immunity has become a focal point in next-generation immunotherapy [34, 35]. The tumor microenvironment, rich in macrophages, dendritic cells, and neutrophils, can be polarized toward either tumor-promoting (M2) or tumoricidal (M1) phenotypes [36]. Novel immunotherapies aim to repurpose tumor-associated macrophages (TAMs) by inhibiting the CSF-1/CSF-1R axis or activating STING and TLR signaling pathways to augment antigen presentation and pro-inflammatory cytokine production [37, 38]. These innate immune-targeted strategies not only enhance T-cell priming but also remodel the TME into a more permissive state for immune infiltration and effector function.

Beyond cellular checkpoints, tumor metabolism has surfaced as a pivotal regulator of immune competence. Enzymes such as indoleamine 2,3-dioxygenase (IDO) deplete tryptophan, generating immunosuppressive metabolites like kynurenine that impair T-cell proliferation [39, 40]. Although first-generation IDO inhibitors yielded limited efficacy in clinical trials, combinatorial regimens and second-generation metabolic modulators continue to hold promise [41, 42]. Furthermore, the adenosine signaling pathway, mediated through CD39/CD73 ectonucleotidases, has been implicated in T-cell anergy within hypoxic TMEs [43, 44]. Therapeutic disruption of this pathway could reactivate immune responses and synergize with existing checkpoint inhibitors.

A particularly transformative domain in next-generation immunotherapy involves adoptive cellular therapies, including chimeric antigen receptor (CAR)-T cells, T-cell receptor (TCR)-engineered lymphocytes, and NK cell-based immunotherapies [45, 46]. While ex vivo CAR-T cell therapy has achieved extraordinary success in hematological malignancies, its application in solid tumors remains constrained by antigen heterogeneity, on-target/off-tumor toxicity, and manufacturing complexity [47, 48]. Recent breakthroughs in in vivo CAR-T cell generation, however, are revolutionizing this field. Instead of relying on laborious ex vivo manipulation, in vivo CAR-T strategies employ lipid nanoparticles (LNPs), viral vectors, or transposon systems to deliver CAR-encoding mRNA or DNA directly to endogenous T cells within patients. Studies demonstrate the feasibility of generating functional CAR-T cells in vivo that retain potent cytotoxicity and anti-tumor efficacy [49–53]. This innovative approach not only reduces production timelines and costs but also enables on-demand, patient-tailored immunotherapy, marking a paradigm shift in the accessibility and scalability of adoptive cell therapy.

In addition, artificial intelligence (AI) and machine learning (ML) are now integral to the discovery and optimization of next-generation immunotherapies. These computational platforms can decipher multi-omic datasets—spanning genomics, proteomics, and radiomics—to identify predictive biomarkers of response, resistance, and toxicity. Deep learning models have been employed to predict neoantigen immunogenicity and T-cell receptor binding specificity with high precision [54, 55]. AI-driven digital pathology tools can quantify tumor-infiltrating lymphocytes (TILs) and spatial immune architectures, providing prognostic insights into checkpoint blockade efficacy [56, 57]. Moreover, reinforcement learning algorithms are enabling the rational design of combination immunotherapy regimens by simulating tumor–immune dynamics. These advances illustrate how computational and biological sciences converge to enhance therapeutic precision and accelerate translational progress in immuno-oncology.

The next generation of cancer immunotherapy must transcend PD-1 and PD-L1 to integrate multi-modal immune activation, metabolic reprogramming, and data-driven personalization. This evolution demands a comprehensive understanding of the molecular crosstalk within the TME, the systemic immune milieu, and tumor-intrinsic resistance circuits. The ongoing convergence of molecular immunology, systems biology, and computational analytics heralds a future in which immunotherapy is not a one-size-fits-all approach but a dynamically adaptive, precisely engineered intervention. The present study is therefore conceived to explore these emerging frontiers—highlighting the mechanistic underpinnings, therapeutic innovations, and translational potential of immunotherapeutic approaches that target pathways beyond PD-1/PD-L1. By delineating the rationale, mechanisms, and prospects of these novel strategies, this work aims to contribute to the evolving landscape of cancer immunotherapy and support the global transition toward more inclusive, durable, and personalized cancer care.

## Mechanisms of resistance to PD-1/PD-L1 blockade

Resistance to PD-1/PD-L1 inhibitors is a multifaceted challenge and is broadly classified into primary (innate) and acquired resistance. Primary resistance reflects a tumor’s pre-existing characteristics that prevent effective immune activation prior to therapy initiation. Factors include low tumor mutational burden (TMB), absence of immunogenic neoantigens, deficient PD-L1 expression, and a non-inflamed TME that impedes T cell infiltration [58]. Acquired resistance, by contrast, emerges during or after therapy and involves adaptive escape mechanisms. Tumors may upregulate alternative immune checkpoints such as CTLA-4, LAG-3, or TIM-3, acquire mutations in interferon signaling pathways, or develop defects in antigen presentation, rendering immune recognition ineffective [59, 60]. Additionally, the recruitment of regulatory T cells (Tregs), myeloid-derived suppressor cells (MDSCs), and M2-polarized macrophages contributes to an immunosuppressive microenvironment that supports tumor persistence and progression [61]. These complex resistance pathways underscore the necessity for multidimensional approaches that can overcome immune escape. Biomarker-driven strategies to predict and monitor resistance are being actively investigated, along with novel agents designed to simultaneously target multiple immune evasion pathways [19, 62, 63]. Moreover, reprogramming the TME to enhance immune cell infiltration and function remains a major research priority.

To circumvent resistance and broaden efficacy, next-generation immunotherapy is increasingly focused on targeting alternative immune checkpoints (e.g., TIGIT, VISTA), activating co-stimulatory pathways (e.g., CD137/4-1BB, OX40, CD40), and mobilizing innate immune effectors such as NK cells and dendritic cells. This is being achieved through approaches like bispecific antibodies, agonist antibodies, and adoptive cell therapies, including CAR-T and CAR-NK cells [64–66]. Additionally, combination regimens involving PD-1/PD-L1 inhibitors and chemotherapy, radiotherapy, or targeted therapies can modulate the TME, enhance antigen release, and amplify immune activation [67]. Personalized neoantigen vaccines and oncolytic viruses are also emerging as innovative platforms to overcome immune resistance by enhancing antigenicity and immunogenicity of tumors [68–70]. Collectively, these integrated strategies anchored in a deeper understanding of tumor–immune system crosstalk hold the potential to reshape the immunotherapy landscape. By tailoring treatment to patient-specific immunogenomic profiles and modulating immune resistance pathways, the next wave of cancer immunotherapy may achieve greater efficacy, broader applicability, and longer-lasting responses across a wide spectrum of malignancies.

## Emerging targets in cancer immunotherapy

### Novel immune checkpoints beyond PD-1/PD-L1

The limitations of PD-1/PD-L1 blockade have catalyzed a surge in research aimed at identifying alternative immune checkpoints that regulate T cell activity and contribute to tumor immune escape. Several emerging targets—including Lymphocyte-Activation Gene 3 (LAG-3), T-cell immunoglobulin and mucin-domain containing-3 (TIM-3), TIGIT, and OX40—are now at the forefront of next-generation immunotherapeutic strategies [32, 71–73]. LAG-3 negatively regulates T cell proliferation and cytokine production and is often co-expressed with PD-1 on exhausted T cells within the tumor microenvironment (TME) [74]. Therapeutic inhibition of LAG-3, alone or in combination with PD-1 inhibitors, has shown potential to reinvigorate exhausted T cells and enhance anti-tumor immunity [75, 76]. TIM-3, another inhibitory receptor, is associated with terminal T cell exhaustion and immune tolerance. Its co-expression with PD-1 on tumor-infiltrating lymphocytes (TILs) correlates with poor clinical outcomes, and dual blockade of TIM-3 and PD-1 has demonstrated synergistic effects in preclinical models [77, 78]. TIGIT is a checkpoint receptor expressed on activated T cells and NK cells that suppresses immune activation through engagement with its ligands CD155 and CD112. Inhibiting TIGIT can reverse T cell dysfunction and restore NK cell-mediated cytotoxicity, making it an appealing target in both solid and hematologic malignancies [31, 79]. In contrast to these inhibitory checkpoints, OX40 (CD134) acts as a co-stimulatory molecule that promotes T cell expansion, effector function, and memory formation [80]. Agonist antibodies targeting OX40 have demonstrated potential to amplify T cell responses and are under evaluation in combination regimens with PD-1 inhibitors [81, 82]. These targets reflect a broader shift toward multi-checkpoint immunotherapy, where modulating multiple inhibitory and stimulatory signals can overcome immune resistance and improve therapeutic outcomes. Multiple clinical trials are currently evaluating these agents as monotherapies or in rational combinations, with early-phase data showing encouraging signals of efficacy and tolerability.

### **Co-**stimulatory molecules enhancing anti-tumor responses

While immune checkpoint blockade focuses on lifting the inhibitory brakes on immune cells, activating co-stimulatory pathways provides the gas pedal needed to enhance and sustain T cell responses. Several key co-stimulatory receptors—4-1BB (CD137), Glucocorticoid-Induced TNFR-Related protein (GITR), and CD40—are under active investigation for their ability to potentiate anti-tumor immunity [83]. 4-1BB, expressed on activated T cells, NK cells, and dendritic cells, is a potent activator of cytotoxic lymphocytes. Engagement of 4-1BB enhances T cell survival, proliferation, and memory formation, leading to durable anti-tumor responses [84]. GITR, upregulated on both effector T cells and regulatory T cells (Tregs), plays a dual role by enhancing effector T cell activity while diminishing Treg-mediated immunosuppression [85]. Agonist antibodies targeting GITR have shown promise in preclinical models and are currently being evaluated in clinical trials for solid tumors and lymphomas [86, 87].

CD40, primarily expressed on antigen-presenting cells (APCs), is critical for T cell priming and dendritic cell activation. CD40 agonists enhance antigen presentation and bridge innate and adaptive immunity by promoting cytokine production and T cell activation [88, 89]. Harnessing co-stimulatory signals in tandem with checkpoint blockade holds promise for synergistic immunotherapy, particularly in tumors with low baseline immune infiltration. These strategies may expand the immunogenicity of “cold” tumors and enable more patients to benefit from immune-based treatments.

### Targeting the innate immune system in immunotherapy

Beyond adaptive immunity, the innate immune system—comprising natural killer (NK) cells, macrophages, and dendritic cells—plays a pivotal role in shaping anti-tumor responses and immunotherapy outcomes. Targeting these components represents a promising frontier in immuno-oncology [90, 91]. NK cells, which recognize and eliminate stressed or transformed cells without prior antigen exposure, are emerging as critical effectors in tumor immunosurveillance. Strategies to enhance NK cell activity include cytokine stimulation (e.g., IL-2, IL-15), checkpoint inhibition (e.g., targeting NKG2A or TIGIT), and adoptive transfer of ex vivo expanded or engineered NK cells. Monoclonal antibodies and bispecific killer cell engagers (BiKEs) are also being developed to promote NK-mediated cytotoxicity [92]. Macrophages, particularly tumor-associated macrophages (TAMs), are plastic cells that can adopt either pro-inflammatory (M1) or immunosuppressive (M2) phenotypes. In cancer, TAMs frequently exhibit an M2-like profile that promotes angiogenesis, suppresses T cell activity, and facilitates tumor growth [93–95]. Therapeutic strategies aim to reprogram TAMs from M2 to M1 phenotype or deplete immunosuppressive TAMs using agents such as CSF1R inhibitors and CD47-SIRPα antagonists, the latter of which disrupts the “don’t eat me” signal used by cancer cells to evade macrophage-mediated phagocytosis [96, 97]. Targeting innate immunity not only boosts anti-tumor responses but may also enhance the priming and recruitment of adaptive immune cells, creating a more favorable tumor immune microenvironment. This is particularly relevant in tumors with low T cell infiltration, where innate immune modulation may serve as a necessary prelude to successful checkpoint inhibition. Integrating innate immune activators with adaptive immune checkpoint therapies represents a holistic approach to cancer immunotherapy, capable of overcoming resistance and expanding efficacy across tumor types.

## Combination therapies to enhance efficacy

### Rationale for combining PD-1/PD-L1 inhibitors with other modalities

While immune checkpoint blockade with PD-1/PD-L1 inhibitors has shown transformative potential [67, 98], monotherapy often fails to elicit durable responses across all cancer types [99, 100]. This has driven a strong rationale for combining checkpoint inhibitors with other therapeutic modalities to synergistically enhance antitumor efficacy, overcome resistance, and expand the population of patients who benefit. A compelling combination strategy involves dual immune checkpoint blockade, particularly co-inhibition of PD-1/PD-L1 and CTLA-4. These molecules regulate distinct stages of the immune response: CTLA-4 acts primarily during T cell priming in lymphoid tissues, whereas PD-1/PD-L1 modulates T cell activity within the tumor microenvironment. Together, their blockade can reinvigorate exhausted T cells, promote effector function, and enhance tumor infiltration—producing more robust and durable responses [101, 102]. Beyond dual checkpoint inhibition, chemotherapy and radiotherapy are being integrated with PD-1/PD-L1 inhibitors due to their immunomodulatory effects. These treatments induce immunogenic cell death (ICD), releasing tumor antigens and danger-associated molecular patterns (DAMPs) that facilitate dendritic cell activation and T cell priming. Additionally, radiation and chemotherapy may upregulate PD-L1 expression or reduce suppressive immune cell populations, thereby sensitizing tumors to checkpoint inhibition [103, 104]. Targeted therapies, especially those modulating oncogenic drivers (e.g., EGFR, BRAF, VEGF), also offer synergy with immunotherapy. These agents can directly inhibit tumor growth while reconditioning the tumor microenvironment, enhancing tumor antigen presentation, vascular normalization, and T cell infiltration, thus improving immune responsiveness. The integration of immunotherapy with multiple modalities represents a multidimensional strategy to address tumor heterogeneity, immunosuppression, and dynamic immune escape.

### Studies on combination immunotherapy

Studies supporting combination immunotherapy is rapidly expanding, with numerous trials investigating how to enhance the therapeutic window of PD-1/PD-L1 blockade through co-administration with immunomodulators, cytotoxic agents, targeted therapies, or radiotherapy. Among the most established combinations is nivolumab (anti–PD-1) with ipilimumab (anti–CTLA-4). In advanced melanoma, this dual blockade has significantly improved progression-free and overall survival compared to monotherapy, and has since been approved in multiple cancer types including renal cell carcinoma and mesothelioma [105]. In non-small cell lung cancer (NSCLC), combinations such as pembrolizumab plus platinum-based chemotherapy (KEYNOTE-189) and atezolizumab with bevacizumab and chemotherapy (IMpower150) have shown superior survival outcomes compared to chemotherapy alone, leading to widespread clinical adoption [106, 107]. Targeted therapies have also been successfully combined with immunotherapy. For example, pembrolizumab plus axitinib, a VEGFR tyrosine kinase inhibitor, has demonstrated improved objective response rates and survival in renal cell carcinoma compared to sunitinib monotherapy [108, 109]. Radiation therapy has gained interest as an immune adjuvant due to its capacity to generate in situ vaccination effects. Radiation-induced tumor cell death can release neoantigens and promote immune cell recruitment. Notably, abscopal effects, where localized radiation triggers systemic antitumor immunity, are increasingly reported when combined with PD-1/PD-L1 blockade [110]. Despite these advances, challenges remain. Some combinations can result in increased immune-related adverse events (irAEs), and not all synergistic effects observed in preclinical models translate into clinical benefit.

### Activating the cancer-immunity cycle

**Fig. 3:**
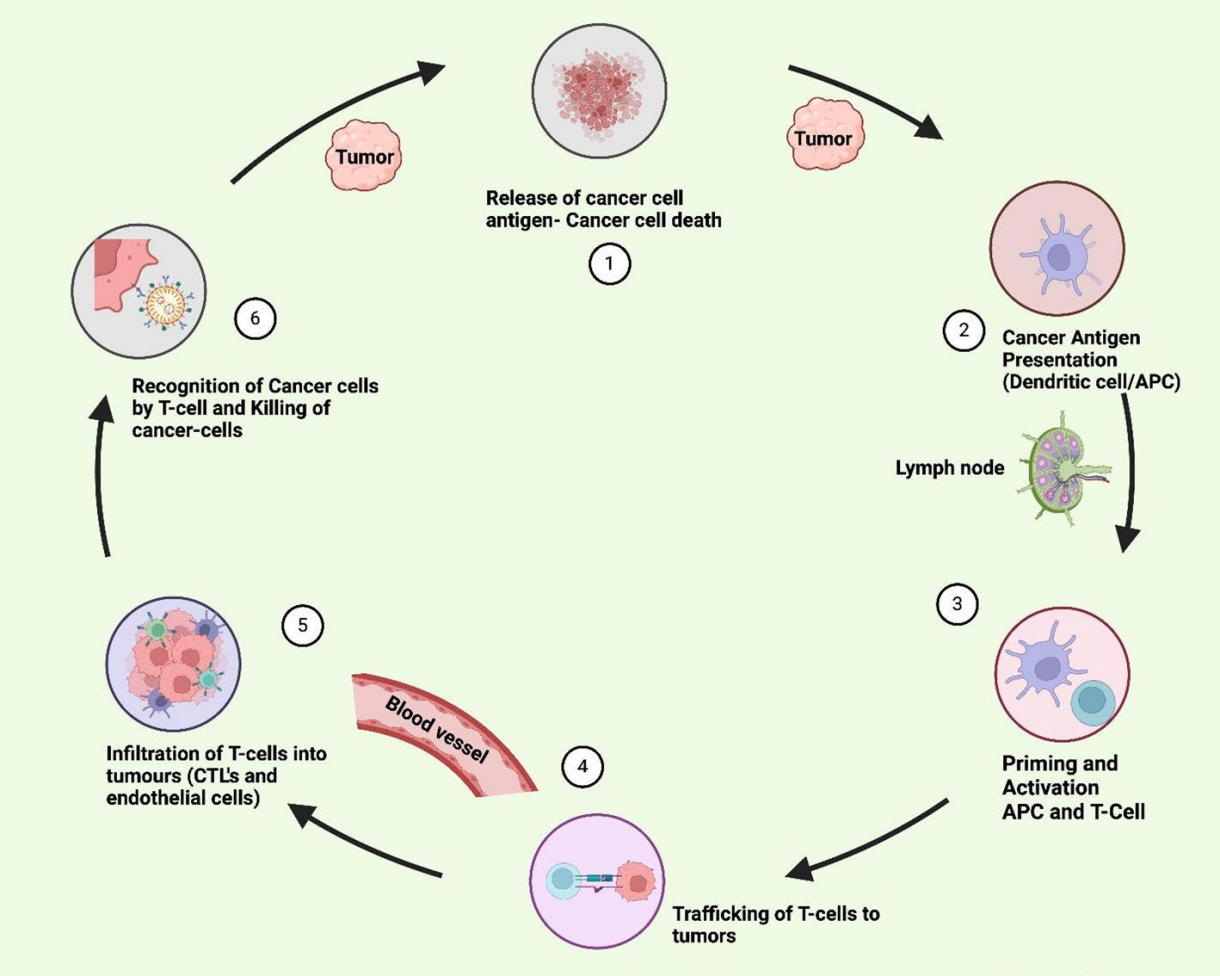
Overview of the cancer-immunity cycle. The cycle includes: **1** release of tumor antigens, **2** antigen presentation by dendritic cells, **3** T-cell priming and activation, **4** T-cell trafficking, **5** infiltration into tumors, and **6** recognition and killing of cancer cells. Successful execution of each step amplifies the anti-tumor immune response

The concept of the cancer–immunity cycle provides a comprehensive framework to understand and enhance anti-tumor immune responses. This cycle includes: (1) release of tumor antigens, (2) antigen presentation, (3) T cell priming and activation, (4) T cell trafficking, (5) infiltration into tumors, (6) tumor recognition and tumor cell killing; each stage offering an opportunity for therapeutic intervention (Fig. 3) [98, 99]. Checkpoint inhibitors such as PD-1/PD-L1 and CTLA-4 blockers primarily act at stages 3 and 6 of the cancer–immunity cycle, where they enhance T-cell activation and effector function by disrupting inhibitory signaling pathways. However, to amplify the entire cycle, combinatorial interventions are necessary. Cancer vaccines and oncolytic viruses can increase the release and presentation of tumor antigens, boosting T cell priming [111]. For instance, oncolytic viruses promote direct tumor lysis while stimulating local inflammation and immune cell recruitment [112, 113]. Targeting the tumor microenvironment (TME) is also critical for reactivating immune responses. Strategies include depletion of suppressive immune cells (e.g., regulatory T cells, MDSCs), normalization of tumor vasculature, and modulation of stromal barriers to facilitate T cell infiltration. Additionally, metabolic reprogramming agents can reverse T cell dysfunction caused by hypoxia, adenosine accumulation, and nutrient depletion within the TME [114]. By orchestrating therapies across the various stages of the immunity cycle rather than relying solely on checkpoint inhibition—a more comprehensive and durable antitumor response can be achieved. Such strategies may also convert immunologically “cold” tumors into “hot” ones, rendering them more susceptible to immunotherapy. This integrative approach, targeting both innate and adaptive immunity, positions the cancer–immunity cycle as a central model for rational combination immunotherapy design. As mechanistic understanding grows, this model will continue to inform therapeutic innovation and trial design, paving the way toward personalized and curative cancer immunotherapy. The effective orchestration of the cancer immunity cycle represents a cornerstone in modern immunotherapy, reinforcing the rationale for multi-targeted strategies [115, 116].

## Personalized and biomarker-driven immunotherapy

### Predictive biomarkers for immunotherapy response

The integration of predictive biomarkers into cancer immunotherapy marks a transformative step toward personalized medicine, allowing for the optimization of treatment strategies tailored to the molecular and immunologic profile of each patient. Biomarkers offer valuable insight into the likelihood of therapeutic response, facilitating more effective treatment decisions while minimizing unnecessary exposure to ineffective therapies. PD-L1 expression remains the most widely studied biomarker for PD-1/PD-L1 blockade, with higher expression levels often correlating with improved clinical outcomes. However, variability in response among PD-L1–positive and negative patients underscores the limitations of this single marker and the need for complementary indicators [117, 118]. Tumor Mutational Burden (TMB) is another critical biomarker that reflects the number of somatic mutations in a tumor’s genome [119, 120]. Tumors with high TMB generate more neoantigens, increasing the likelihood of immune recognition and response to checkpoint inhibitors [121]. Similarly, microsatellite instability-high (MSI-H) and mismatch repair deficiency (dMMR) have demonstrated strong predictive value in cancers such as colorectal, endometrial, and gastric cancers, identifying patients who are highly responsive to immunotherapy [122, 123]. Emerging immune gene signatures, derived from transcriptomic profiling, capture the complexity of the tumor-immune landscape and provide predictive insights beyond traditional markers. These signatures reflect immune cell infiltration, activation states, and inflammatory cytokine profiles. Additionally, liquid biopsies, particularly through the detection of circulating tumor DNA (ctDNA), offer a non-invasive means of tracking tumor dynamics and treatment responses in real-time [124, 125]. These tools support adaptive therapy strategies based on evolving tumor characteristics. Collectively, these biomarkers are guiding a shift from a one-size-fits-all paradigm to a data-driven, patient-specific approach in cancer immunotherapy enhancing efficacy, reducing toxicity, and improving clinical outcomes.

### Emerging biomarkers beyond PD-L1

Beyond PD-L1, a broader set of emerging biomarkers is being explored to capture the diverse biological factors influencing immunotherapy outcomes. Among them, TMB, MSI, and immune gene signatures have gained particular attention for their predictive potential and mechanistic relevance [126]. Tumor Mutational Burden (TMB) quantifies the total number of somatic mutations within tumor coding regions. High TMB correlates with a greater likelihood of generating immunogenic neoantigens, rendering tumors more susceptible to immune attack. Clinical evidence consistently links elevated TMB with favorable responses to PD-1/PD-L1 blockade across multiple tumor types, including melanoma and NSCLC [127]. Microsatellite instability (MSI) and deficient mismatch repair (dMMR), resulting from defects in DNA repair mechanisms, lead to high levels of mutations and neoantigen production [128]. MSI-H/dMMR tumors have shown exceptional responsiveness to checkpoint inhibitors, leading to FDA approvals for immunotherapy across solid tumors based on these markers, irrespective of tumor origin [129, 130]. Immune gene expression signatures, such as interferon-gamma–related signatures or T-effector cell profiles, serve as proxies for T cell inflammation and tumor immunogenicity. These gene panels distinguish “hot” tumors—those with pre-existing immune infiltration from “cold” tumors that are less responsive to immunotherapy [131]. Such stratification supports informed decisions regarding treatment escalation, conversion strategies, or alternative modalities. The continued evolution and validation of these biomarkers are critical to advancing precision immuno-oncology. By integrating multi-parametric data—including genomic, transcriptomic, and proteomic features—oncologists can make more accurate patient selections and design tailored therapeutic regimens, improving outcomes across a broader spectrum of malignancies.

### Role of next-generation sequencing and liquid biopsies

Technological advances in next-generation sequencing (NGS) and liquid biopsies have revolutionized personalized immunotherapy by enabling a deeper understanding of tumor biology and immune dynamics with minimally invasive sampling. NGS allows for comprehensive genomic profiling, detecting mutations, gene fusions, copy number variations, and mutational signatures across the tumor genome. This facilitates identification of high TMB, MSI status, neoantigen load, and actionable oncogenic drivers—all of which influence immunotherapy responsiveness. For example, NGS-derived TMB scores are now incorporated into FDA-approved companion diagnostics for certain immune checkpoint inhibitors [132]. Additionally, NGS can uncover resistance mechanisms, such as mutations in beta-2 microglobulin (B2M), JAK/STAT pathway disruptions, or interferon signaling defects, which may predict immune escape and treatment failure [129, 133]. Thus, genomic profiling not only informs treatment selection but also guides dynamic therapy adjustments. Liquid biopsies, through analysis of circulating tumor DNA (ctDNA), tumor cells, and exosomes, offer a real-time window into tumor evolution and therapeutic response [125]. These assays can detect emerging resistance, measure minimal residual disease (MRD), and monitor immune-related genomic changes. Their non-invasive nature makes them ideal for serial monitoring and rapid clinical decision-making. The integration of NGS and liquid biopsy platforms empowers oncologists to implement truly personalized immunotherapy, where treatment regimens are continuously adapted to tumor evolution. These tools are instrumental in identifying the right therapy for the right patient at the right time, offering a significant step forward in precision oncology.

## Novel approaches in immunotherapy delivery

### Innovations in immunotherapy delivery systems

Advancements in immunotherapy delivery are revolutionizing cancer treatment by improving therapeutic precision, reducing systemic toxicity, and enhancing antitumor efficacy. Novel delivery systems aim to maximize immune activation at tumor sites while minimizing off-target effects and treatment-related toxicity. Localized delivery strategies, such as implantable devices for sustained drug release or direct intratumoral injections, are gaining traction for improving therapeutic index. These techniques enable concentration of immune agents at the tumor microenvironment, overcoming barriers posed by immune-excluded or immunosuppressive tumors. Additionally, nanoparticle-based systems—including liposomes, dendrimers, and polymeric nanoparticles—allow for targeted delivery of cytokines, immune checkpoint inhibitors, or tumor antigens, enhancing bioavailability and tumor selectivity [134, 135]. Similarly, viral vectors have been harnessed for precise delivery of immunomodulatory genes directly to tumors [136]. Emerging bispecific antibodies designed to simultaneously bind tumor-associated antigens and immune effector molecules like CD3 on T cells are a particularly promising innovation [137, 138]. These antibodies physically bridge T cells with cancer cells, enhancing synapse formation and cytotoxic activity while maintaining immune selectivity. By targeting both tumor antigens and T cell activation pathways, bispecifics induce robust immune responses with minimal damage to surrounding healthy tissues [139]. Another major breakthrough in delivery innovation is engineered immune cells, particularly Chimeric Antigen Receptor (CAR) T cells, which involve modifying a patient’s T cells to express synthetic receptors targeting tumor-specific antigens [140]. Once reinfused, these modified T cells exhibit enhanced recognition, proliferation, and cytotoxicity against cancer cells [141, 142]. While CAR-T therapies have shown remarkable success in hematologic malignancies, their application is expanding to include CAR-NK cells and TCR-engineered T cells for broader cancer types, including solid tumors [143, 144]. Together, these advances in delivery systems represent transformational solutions to challenges in cancer immunotherapy—particularly limited accessibility, suboptimal responses in solid tumors, and immune-related adverse events. Continued innovation will extend the reach and effectiveness of immunotherapies, enabling more personalized and effective treatment paradigms.

### Oncolytic viruses as cancer immunotherapy agents

Oncolytic virotherapy harnesses viruses that selectively infect and destroy cancer cells while simultaneously stimulating the host’s immune system to mount an antitumor response. Either naturally occurring or genetically engineered, these viruses exploit defective antiviral mechanisms in tumor cells, allowing selective replication and oncolysis while sparing normal tissues [145]. Upon infection, oncolytic viruses (OVs) induce tumor cell lysis, releasing tumor-associated antigens (TAAs) and danger-associated molecular patterns (DAMPs). This converts the tumor into an in-situ vaccine, triggering dendritic cell activation, cytokine release, and recruitment of effector immune cells, including cytotoxic T lymphocytes and NK cells [146]. Furthermore, OVs reshape the tumor microenvironment (TME) by promoting pro-inflammatory cytokine secretion (e.g., IL-12, GM-CSF) and reducing immunosuppressive elements such as regulatory T cells and myeloid-derived suppressor cells [147]. These immunogenic effects enhance antigen presentation and facilitate systemic immune activation against metastatic or non-injected lesions, contributing to abscopal effects. Recent engineering efforts focus on arming OVs with immunostimulatory genes, such as cytokines, checkpoint inhibitors, or co-stimulatory ligands, to amplify immune responses. Notable clinical progress has been observed with agents like T-VEC (talimogene laherparepvec) in melanoma, and several next-generation OVs are under investigation in solid tumors [148]. OVs represent a dual-function platform combining direct oncolysis with immune activation—and hold immense promise as monotherapy or in combination with immune checkpoint inhibitors and adoptive cell therapy. Their ability to transform immunologically “cold” tumors into “hot” tumors places them at the forefront of oncoimmunology research and clinical translation.

### Personalized neoantigen vaccines

Neoantigen-based vaccines represent a cutting-edge strategy in personalized cancer immunotherapy, targeting tumor-specific mutations that generate novel peptide antigens [68, 149]. Unlike shared tumor-associated antigens, neoantigens arise from individual somatic mutations and are absent from normal tissues, minimizing the risk of off-target toxicity [150]. The vaccine development process begins with tumor and normal DNA sequencing to identify non-synonymous mutations. In silico algorithms predict which mutated peptides are likely to bind major histocompatibility complex (MHC) molecules and elicit T cell responses [151]. These predicted neoepitopes are synthesized and formulated into peptide, RNA, or dendritic cell–based vaccines. Upon administration, the vaccine primes neoantigen-specific cytotoxic T lymphocytes (CTLs), capable of recognizing and eliminating tumor cells expressing these unique mutations.

Neoantigen vaccines have demonstrated feasibility, safety, and immunogenicity in early-phase trials across multiple cancers, including melanoma, glioblastoma, and NSCLC [121, 152–154]. Importantly, their personalized design allows immune responses to be tailored to the mutational landscape of each patient, offering an unprecedented level of specificity.

Beyond efficacy, neoantigen vaccines can address tumor heterogeneity and antigen escape, targeting multiple epitopes simultaneously and evolving with the tumor’s mutational profile. Future efforts are directed at improving neoantigen prediction accuracy, optimizing vaccine delivery platforms, and combining vaccines with checkpoint blockade or other immunotherapies to enhance potency and durability. With advancing genomics, computational biology, and immunologic profiling, neoantigen vaccines are poised to become a foundational element of personalized immuno-oncology, offering custom-designed, mutation-targeted immunotherapies with high precision and minimal off-target effects.

## Overcoming the tumor microenvironment barrier

### Immunosuppressive tumor microenvironment: a major obstacle

The tumor microenvironment (TME) constitutes a complex, dynamic milieu that significantly hampers the effectiveness of cancer immunotherapy. Comprising malignant cells, stromal components, blood vessels, extracellular matrix (ECM), and infiltrating immune cells, the TME supports tumor progression and mediates immune evasion through a multitude of mechanisms [155]. A hallmark of the TME is its immunosuppressive cellular composition, including regulatory T cells (Tregs), myeloid-derived suppressor cells (MDSCs), and tumor-associated macrophages (TAMs) of the M2 phenotype [156]. These cell populations actively inhibit effector T cells and NK cells via the secretion of anti-inflammatory cytokines such as TGF-β and IL-10, competition for metabolic resources, and upregulation of inhibitory ligands like PD-L1 [156, 157]. Hypoxia, a characteristic feature of rapidly growing tumors, further compounds immunosuppression by stabilizing HIF-1α, increasing PD-L1 expression, and limiting T cell infiltration and function [158]. Concurrently, the dense extracellular matrix (ECM) and aberrant tumor vasculature act as physical and biochemical barriers that restrict immune cell trafficking and drug delivery [159]. Moreover, the metabolic reprogramming within the TME—characterized by high glycolytic flux, nutrient depletion (e.g., glucose, tryptophan), and accumulation of immunosuppressive metabolites such as adenosine, lactate, and kynurenine—further impairs immune effector function [114, 160]. Understanding and overcoming these barriers of the TME is essential for enhancing the efficacy of cancer immunotherapy. Emerging strategies target these suppressive networks to reshape the TME into a more immunologically permissive state, thereby enabling more durable and effective anti-tumor responses [161, 162].

### Strategies to modulate the tumor microenvironment

Modulating the immunosuppressive tumor microenvironment is a critical objective in improving immunotherapeutic outcomes. Several targeted strategies are being investigated to reprogram the TME and restore effective immune surveillance. Enzymatic degradation of ECM components such as with hyaluronidase has shown promise in facilitating T cell access to tumor cores and improving drug perfusion [163]. Modulating ECM stiffness and composition can also recondition the TME to become more immuno-responsive [164]. TAMs, particularly of the M2 phenotype, support tumor growth and immune suppression. Therapeutic strategies include CSF1R inhibitors to deplete TAMs or CD40 agonists to reprogram them toward a pro-inflammatory M1 phenotype, enhancing their antigen-presenting function and cytotoxic potential [165]. MDSCs suppress T cell activation and are recruited by tumors to dampen immune responses [166]. Approaches to reduce their impact include all-trans retinoic acid (ATRA) to differentiate MDSCs into non-suppressive phenotypes and PI3Kδ inhibitors to impair their signaling pathways. Additionally, CCR2 blockade can disrupt MDSC recruitment to the tumor site [167]. These interventions aim to disarm the immunosuppressive elements of the TME, improve T cell trafficking, and potentiate immune checkpoint blockade. As our understanding deepens, combining TME-modulating agents with immunotherapy holds the potential to achieve more durable and universal responses across diverse tumor types.

### Gut microbiota and its impact on immunotherapy outcomes

The gut microbiota has emerged as a pivotal determinant of cancer immunotherapy efficacy. Comprising trillions of microorganisms, this microbial ecosystem plays a crucial role in shaping systemic immunity and modulating host responses to immune checkpoint inhibitors (ICIs). Recent studies have demonstrated that specific microbial compositions are associated with enhanced responses to PD-1/PD-L1 blockade [168–170]. Beneficial bacteria such as *Bifidobacterium*, *Akkermansia muciniphila*, and *Faecalibacterium prausnitzii* have been correlated with improved therapeutic outcomes, likely through mechanisms involving dendritic cell maturation, T cell priming, and attenuation of immunosuppressive pathways [171, 172]. Conversely, disruption of the gut microbiome, notably through the use of broad-spectrum antibiotics, has been linked to diminished ICI efficacy, underscoring the need for careful antimicrobial stewardship in patients undergoing immunotherapy [173, 174]. Interventions to enhance microbiota composition include:

- Probiotics and prebiotics to selectively promote beneficial microbial strains,
- Fecal microbiota transplantation (FMT) from ICI responders to non-responders,
- Dietary modulation to promote a favorable microbial milieu.

These approaches aim to establish a microbiome-immune axis conducive to effective antitumor responses. The integration of microbiome profiling and personalized microbial interventions represent a novel frontier in optimizing immunotherapy.

As our understanding of the gut–immune–tumor axis continues to evolve, it holds the potential to become a critical adjunct in cancer immunotherapy, opening new avenues for enhancing efficacy, reducing toxicity, and personalizing treatment strategies. Future studies are expected to refine microbial profiling to personalize immunotherapy protocols, potentially incorporating microbiota modulation into standard cancer care.

## Challenges and future perspectives

### Barriers to broader immunotherapy adoption

Despite the transformative impact of immunotherapy in oncology, several critical challenges hinder its broad implementation across clinical settings. One of the most pressing concerns is the emergence of immune-related adverse events (irAEs) [175]. These effects, which can affect multiple organ systems, result from the heightened immune activation induced by therapies such as checkpoint inhibitors and CAR T-cell treatments. Effective management of these toxicities requires precise diagnostic strategies, accurate grading of severity, and the cautious use of immunosuppressive agents. However, such interventions must be finely balanced to avoid compromising the desired antitumor immunity. Research is increasingly focused on the identification of predictive biomarkers for toxicity, which could allow clinicians to pre-emptively tailor immunotherapy regimens to individual risk profiles, enhancing safety without reducing efficacy.

A parallel and equally formidable barrier is the economic burden associated with advanced immunotherapies, particularly autologous treatments such as CAR T-cell therapy [176]. These therapies demand complex manufacturing pipelines, including cell harvesting, genetic modification, ex vivo expansion, and reinfusion, in addition to post-treatment monitoring. The resulting costs place these innovations beyond the reach of many healthcare systems, especially in resource-limited settings. In response, efforts are now underway to optimize manufacturing efficiency, including the development of automated, decentralized production models. Furthermore, alternative reimbursement frameworks and policy reforms are being proposed to ensure broader access to these life-saving therapies. Beyond financial constraints, systemic limitations within healthcare infrastructure further impede access. The administration of immunotherapies requires specialized facilities, highly trained personnel, and robust monitoring capabilities to manage complex toxicities. These demands are often unmet in developing regions, underscoring the need for strategic investments in capacity building, cross-sector collaborations, and the global diffusion of immunotherapy expertise.

Clinical heterogeneity also poses a significant hurdle. Patient-specific variability in immunotherapy response remains poorly understood and often leads to unpredictable outcomes. The biological basis for this variability includes factors such as the tumor microenvironment, tumor mutational burden, immune infiltration, and even gut microbiota composition. Tackling this complexity necessitates the design of rational combination regimens, development of robust predictive biomarkers, and expansion of clinical trials to include diverse populations and tumor types. To overcome these barriers and democratize access to immunotherapy, a comprehensive, multi-tiered strategy is essential. This includes advancing translational research to elucidate resistance mechanisms, driving innovation in delivery and production technologies, enhancing safety profiles, and expanding healthcare infrastructure. The future of immunotherapy rests not only on scientific breakthroughs, but on the integration of innovation, policy, and equitable access across the global oncology landscape.

### Role of artificial intelligence and machine learning in advancing immunotherapy

The convergence of artificial intelligence (AI) and machine learning (ML) with immuno-oncology is redefining the frontiers of precision medicine [177]. What once seemed a futuristic vision is now rapidly becoming clinical reality. By harnessing vast multi-dimensional datasets—including genomic, transcriptomic, proteomic, radiomic, and clinical data—AI and ML models can uncover hidden biological patterns that are often imperceptible to human analysis [178]. These computational tools are not only capable of decoding the molecular underpinnings of tumor–immune interactions but also of generating actionable insights that drive individualized treatment strategies [179].

Deep learning algorithms have demonstrated remarkable accuracy in predicting neoantigen presentation and T-cell receptor specificity, thereby accelerating the design of personalized cancer vaccines and adoptive cell therapies [55, 180]. Likewise, AI-powered digital pathology is revolutionizing diagnostic oncology by quantifying tumor-infiltrating lymphocytes (TILs) and mapping spatial immune architectures from whole-slide images—offering reliable biomarkers for predicting PD-1/PD-L1 checkpoint inhibitor responses [181, 182]. In parallel, reinforcement learning frameworks are being leveraged to model dynamic tumor–immune interactions, thereby optimizing combination regimens involving checkpoint inhibitors, targeted agents, and conventional therapies such as chemotherapy and radiotherapy [183–185]. Through such adaptive modeling, AI minimizes empirical trial-and-error in clinical decision-making, bringing precision and speed to therapeutic optimization.

The influence of AI extends far beyond the clinic into the realm of drug discovery and translational research. AI-guided molecular screening accelerates the identification of novel immune targets, shortens vaccine design timelines, and informs the rational engineering of T-cell and CAR-T therapies. In neoantigen discovery, AI has enabled the development of personalized cancer vaccines that precisely engage the immune system with tumor-specific mutations, marking a paradigm shift toward fully individualized immunotherapy [68]. Furthermore, as AI systems evolve, their applications are expanding into decoding the microbiome–immune axis, predicting immune-related adverse events, and even guiding nanotechnology- and CRISPR-Cas9–based therapeutic delivery systems.

In essence, the integration of AI and ML into immuno-oncology represents a pivotal moment in modern cancer research—one where computation meets clinical intuition. By enhancing the precision, adaptability, and scalability of immunotherapeutic strategies, these technologies are transforming cancer immunotherapy from a specialized intervention into a globally accessible, dynamically responsive, and continuously learning treatment paradigm.

### Emerging breakthrough: in vivo CAR-T cell generation

Recent breakthroughs in in vivo CAR-T cell production are redefining the paradigm of adoptive cellular immunotherapy and addressing many of the limitations associated with traditional ex vivo manufacturing. Conventional CAR-T therapy requires the extraction, genetic modification, and ex vivo expansion of patient-derived T cells—a complex, time-consuming, and costly process that restricts accessibility and scalability [47, 186]. In contrast, in vivo CAR-T technology enables the generation of CAR-T cells directly within the patient’s body, bypassing the need for external cell manipulation [52, 187, 188]. This is achieved through lipid nanoparticles (LNPs), viral vectors, or transposon-based systems that deliver CAR-encoding mRNA or DNA directly to circulating T cells, inducing their transformation into functional CAR-T cells in situ [187, 189, 190].

Studies have demonstrated the feasibility and therapeutic potency of this approach, showing that in vivo–generated CAR-T cells can mount robust and durable anti-tumor immune responses comparable to or even surpassing traditional methods [49, 188]. Importantly, this technology minimizes production time, reduces manufacturing costs, and enables rapid, scalable deployment—potentially making CAR-T therapies more accessible to a broader patient population. When combined with AI-driven predictive modeling and optimization algorithms, in vivo CAR-T platforms hold the promise of real-time design, monitoring, and adjustment of therapeutic constructs, ushering in a new generation of personalized, adaptive, and cost-effective immunotherapies.

## Conclusion

Cancer immunotherapy has transformed the landscape of oncologic treatment, particularly through the clinical success of PD-1/PD-L1 inhibitors, which have yielded durable responses and improved survival across several malignancies. However, the variability in patient responses and the pervasive issue of therapeutic resistance underscore a pressing need to broaden the immunotherapeutic arsenal. The path forward must transcend the current checkpoint blockade paradigm by exploring novel immune targets, developing personalized neoantigen-based vaccines, refining engineered cellular therapies, and integrating immunotherapy with other treatment modalities such as chemotherapy, radiotherapy, and targeted agents. Such diversification is imperative to extend the benefits of immunotherapy to a wider spectrum of tumor types and patient populations particularly those who have thus far demonstrated limited or no response. By embracing personalized strategies informed by predictive biomarkers, next-generation sequencing, and real-time disease monitoring, we can transition toward a truly tailored model of cancer care. This evolving paradigm holds the potential not only to enhance therapeutic outcomes but to ultimately reposition cancer as a manageable, chronic condition.

The future of cancer immunotherapy is undeniably promising. It is being propelled by a global network of researchers, clinicians, and patients committed to innovation and translational impact. Their collective efforts are unraveling the complexities of immune–tumor interactions, fostering the discovery of more precise and durable interventions, and challenging the limits of what immunotherapy can achieve. In this dynamic landscape, the oncology community is called upon to sustain its momentum by cultivating collaboration across disciplines, advancing clinical trial design, and prioritizing equitable access to immunotherapeutic advances. As we stand on the cusp of a new era in oncology, the continued evolution of immunotherapy offers not only scientific progress but renewed hope for millions of patients worldwide. It is both a call to action and a reaffirmation of our commitment to conquering cancer through the power of the immune system.

## Data Availability

No datasets were generated or analysed during the current study.

## Declarations

### Dual publication

This manuscript is original, has not been published previously, and is not under consideration for publication elsewhere.

## References

[1] Cao Y, et al. Immune checkpoint molecules in natural killer cells as potential targets for cancer immunotherapy. Signal Transduct Target therapy. 2020;5(1):250. 10.1038/s41392-020-00348-8 PMC7596531 33122640

[2] Janelle V, et al. T-cell immunotherapies targeting histocompatibility and tumor antigens in hematological malignancies. Front Immunol. 2020;11:276. 32153583 10.3389/fimmu.2020.00276 PMC7046834

[3] Cavalluzzo B, et al. Cross-reactive CD8 + T cell responses to tumor-associated antigens (TAAs) and homologous microbiota-derived antigens (MoAs). J Experimental Clin Cancer Res. 2024;43(1):87. 10.1186/s13046-024-03004-z PMC10953141 38509571

[4] Wolf NK, Kissiov DU, Raulet DH. Roles of natural killer cells in immunity to cancer, and applications to immunotherapy. Nat Rev Immunol. 2023;23(2):90–105. 35637393 10.1038/s41577-022-00732-1

[5] Goldmann O, Nwofor OV, Chen Q, Medina E. Mechanisms underlying immunosuppression by regulatory cells. Front Immunol. 2024;15:1328193. 38380317 10.3389/fimmu.2024.1328193 PMC10876998

[6] Nishida N. Role of oncogenic pathways on the cancer immunosuppressive microenvironment and its clinical implications in hepatocellular carcinoma. Cancers. 2021;13(15):3666. 34359568 10.3390/cancers13153666 PMC8345137

[7] Borroni EM, Grizzi F. *Cancer Immunoediting and beyond in 2021*. 2021, MDPI. p. 13275. 10.3390/ijms222413275 PMC8703961 34948072

[8] Roerden M, Spranger S. Cancer immune evasion, immunoediting and intratumour heterogeneity. Nat Rev Immunol. 2025;25(5):353–69. 39748116 10.1038/s41577-024-01111-8

[9] Amin T, et al. Immunoediting Dynamics in Glioblastoma: Implications for Immunotherapy Approaches. Cancer Control. 2024;31:10732748241290067. 39353594 10.1177/10732748241290067 PMC11459535

[10] Marei HE, Hasan A, Pozzoli G, Cenciarelli C. Cancer immunotherapy with immune checkpoint inhibitors (ICIs): potential, mechanisms of resistance, and strategies for reinvigorating T cell responsiveness when resistance is acquired. Cancer Cell Int. 2023;23(1):64. 37038154 10.1186/s12935-023-02902-0 PMC10088229

[11] Lu D, et al. Beyond T cells: understanding the role of PD-1/PD‐L1 in tumor‐associated macrophages. J Immunol Res. 2019;2019(1):1919082. 31781673 10.1155/2019/1919082 PMC6875348

[12] Ai L, Xu A, Xu J. *Roles of PD-1/PD-L1 pathway: signaling, cancer, and beyond.* Regulation of cancer Immune checkpoints: Molecular and cellular mechanisms and therapy, 2020: pp. 33–59. 10.1007/978-981-15-3266-5_3 32185706

[13] Naimi A, et al. Tumor immunotherapies by immune checkpoint inhibitors (ICIs); the pros and cons. Cell Communication Signal. 2022;20(1):44. 10.1186/s12964-022-00854-y PMC8991803 35392976

[14] Sun L, et al. Overall survival, treatment duration, and rechallenge outcomes with ICI therapy for recurrent or metastatic HNSCC. JAMA Netw Open. 2024;7(8):e2428526–2428526. 39158913 10.1001/jamanetworkopen.2024.28526 PMC11333980

[15] Dall’Olio FG, et al. Tumour burden and efficacy of immune-checkpoint inhibitors. Nat reviews Clin Oncol. 2022;19(2):75–90. 10.1038/s41571-021-00564-3 34642484

[16] Mountzios G, et al. Immune-checkpoint inhibition for resectable non-small-cell lung cancer—opportunities and challenges. Nat Reviews Clin Oncol. 2023;20(10):664–77. 10.1038/s41571-023-00794-7 37488229

[17] Belluomini L. *Exploring the Complex Interplay between Myeloid-Derived Suppressor Cells (MDSCs), Interleukins, and Immune Checkpoint Inhibitors (ICIs) in Non-Small Cell Lung Cancer (NSCLC).* 2024.

[18] Gill GS et al. Immune checkpoint inhibitors and immunosuppressive tumor microenvironment: current challenges and strategies to overcome resistance. Immunopharmacol Immunotoxicol, 2025: pp. 1–23. 10.1080/08923973.2025.2504906 40376861

[19] Wang H, et al. Immunotherapy resistance in non-small cell lung cancer: from mechanisms to therapeutic opportunities. J Experimental Clin Cancer Res. 2025;44(1):250. 10.1186/s13046-025-03519-z PMC12374485 40849659

[20] Khosravi GR, et al. Immunologic tumor microenvironment modulators for turning cold tumors hot. Cancer Commun. 2024;44(5):521–53. 10.1002/cac2.12539 PMC11110955 38551889

[21] Ouyang P, et al. Overcoming cold tumors: a combination strategy of immune checkpoint inhibitors. Front Immunol. 2024;15:1344272. 38545114 10.3389/fimmu.2024.1344272 PMC10965539

[22] Hu A, et al. Harnessing innate immune pathways for therapeutic advancement in cancer. Signal Transduct Target Therapy. 2024;9(1):68. 10.1038/s41392-024-01765-9 PMC10961329 38523155

[23] Almawash S. Revolutionary Cancer Therapy for Personalization and Improved Efficacy: Strategies to Overcome Resistance to Immune Checkpoint Inhibitor Therapy. Cancers. 2025;17(5):880. 40075727 10.3390/cancers17050880 PMC11899125

[24] Xiang S, Li S, Xu J. Unravelling T cell exhaustion through co-inhibitory receptors and its transformative role in cancer immunotherapy. Clin Translational Med. 2025;15(5):e70345. 10.1002/ctm2.70345 PMC12104568 40415479

[25] Luo J, et al. Tim-3 pathway dysregulation and targeting in sepsis-induced immunosuppression. Eur J Med Res. 2024;29(1):583. 39696711 10.1186/s40001-024-02203-w PMC11656820

[26] Ciraolo E, et al. Simultaneous genetic ablation of PD-1, LAG-3, and TIM-3 in CD8 T cells delays tumor growth and improves survival outcome. Int J Mol Sci. 2022;23(6):3207. 35328630 10.3390/ijms23063207 PMC8955581

[27] Meng X, Luo Y, Cui L, Wang S. Involvement of Tim-3 in maternal-fetal tolerance: a review of current understanding. Int J Biol Sci. 2025;21(2):789. 39781467 10.7150/ijbs.106115 PMC11705645

[28] Rezazadeh-Gavgani E et al. *Immune Checkpoint Molecules: A Review on Pathways and Immunotherapy Implications.* Immunity, Inflammation and Disease, 2025. 13(4): p. e70196. 10.1002/iid3.70196 PMC12004596 40243372

[29] Kreidieh FY, Tawbi HA. The introduction of LAG-3 checkpoint blockade in melanoma: immunotherapy landscape beyond PD-1 and CTLA-4 inhibition. Therapeutic Adv Med Oncol. 2023;15:17588359231186027. 10.1177/17588359231186027 PMC10357068 37484526

[30] Li Y, et al. Advancement of anti-LAG‐3 in cancer therapy. FASEB J. 2023;37(11):e23236. 37846808 10.1096/fj.202301018R

[31] Zhang P, et al. Targeting TIGIT for cancer immunotherapy: recent advances and future directions. Biomark Res. 2024;12(1):7. 38229100 10.1186/s40364-023-00543-z PMC10790541

[32] Zaheer S, Sureka N. Unlocking new frontiers: novel immune targets for next-generation cancer immunotherapy. Korean J Clin Oncol. 2025;21(2):47. 40916400 10.14216/kjco.24322 PMC12415433

[33] Prakash K, et al. Natural Killer Cell and Extracellular Vesicle-Based Immunotherapy in Thyroid Cancer: Advances, Challenges, and Future Perspectives. Cells. 2025;14(14):1087. 40710340 10.3390/cells14141087 PMC12293766

[34] Mandracci G, Soliman N, Khawanky NE. Overcoming Immune Therapy Resistance in Cancer Through Innate Immune Reprogramming. Int J Mol Sci. 2025;26(19):9554. 41096817 10.3390/ijms26199554 PMC12525178

[35] Zimmermannova O, Caiado I, Ferreira AG, Pereira C-F. Cell fate reprogramming era cancer immunotherapy Front Immunol. 2021;12:714822. 34367185 10.3389/fimmu.2021.714822 PMC8336566

[36] Zhang Q, Sioud M. Tumor-associated macrophage subsets: shaping polarization and targeting. Int J Mol Sci. 2023;24(8):7493. 37108657 10.3390/ijms24087493 PMC10138703

[37] Cao J, Liu C. Mechanistic studies of tumor-associated macrophage immunotherapy. Front Immunol. 2024;15:1476565. 39403370 10.3389/fimmu.2024.1476565 PMC11472702

[38] Khan SU, et al. Reprogramming tumor-associated macrophages as a unique approach to target tumor immunotherapy. Front Immunol. 2023;14:1166487. 37138860 10.3389/fimmu.2023.1166487 PMC10149956

[39] Stone TW, Williams RO. Modulation of T cells by tryptophan metabolites in the kynurenine pathway. Trends Pharmacol Sci. 2023;44(7):442–56. 37248103 10.1016/j.tips.2023.04.006

[40] Fiore A, Murray PJ. Tryptophan and indole metabolism in immune regulation. Curr Opin Immunol. 2021;70:7–14. 33418116 10.1016/j.coi.2020.12.001

[41] Devaraji M, Varghese B, Cheriyan. Immune-based cancer therapies: mechanistic insights, clinical progress, and future directions. J Egypt Natl Cancer Inst. 2025;37(1):62. 10.1186/s43046-025-00319-6 PMC13313453 41016975

[42] Joshi DC, et al. Novel therapeutic agents in clinical trials: emerging approaches in cancer therapy. Discover Oncol. 2024;15(1):342. 10.1007/s12672-024-01195-7 PMC11317456 39127974

[43] Churov A, Zhulai G. Targeting adenosine and regulatory T cells in cancer immunotherapy. Hum Immunol. 2021;82(4):270–8. 33610376 10.1016/j.humimm.2020.12.005

[44] Jiang X, et al. The ectonucleotidases CD39 and CD73 on T cells: The new pillar of hematological malignancy. Front Immunol. 2023;14:1110325. 36776866 10.3389/fimmu.2023.1110325 PMC9911447

[45] Moscarelli J, Zahavi D, Maynard R, Weiner LM. The next generation of cellular immunotherapy: chimeric antigen receptor-natural killer cells. Transplantation Cell therapy. 2022;28(10):650–6. 10.1016/j.jtct.2022.06.025 PMC9547868 35788086

[46] Yu T, et al. Revolution of CAR engineering for next-generation immunotherapy in solid tumors. Front Immunol. 2022;13:936496. 35903099 10.3389/fimmu.2022.936496 PMC9315443

[47] Pinto E, et al. From ex vivo to in vivo chimeric antigen T cells manufacturing: new horizons for CAR T-cell based therapy. J translational Med. 2025;23(1):10. 10.1186/s12967-024-06052-3 PMC11700462 39755643

[48] Zhang G et al. Current Advances and Challenges in CAR-T Therapy for Hematological and Solid Tumors. ImmunoTargets Therapy, 2025: pp. 655–80. 10.2147/ITT.S519616 PMC12212355 40599347

[49] Si X, Xiao L, Brown CE, Wang D. Preclinical evaluation of CAR T cell function: in vitro and in vivo models. Int J Mol Sci. 2022;23(6):3154. 35328572 10.3390/ijms23063154 PMC8955360

[50] Flugel CL, et al. Overcoming on-target, off-tumour toxicity of CAR T cell therapy for solid tumours. Nat Reviews Clin Oncol. 2023;20(1):49–62. 10.1038/s41571-022-00704-3 PMC10278599 36418477

[51] Bandara V, et al. Pre-clinical validation of a pan-cancer CAR-T cell immunotherapy targeting nfP2X7. Nat Commun. 2023;14(1):5546. 37684239 10.1038/s41467-023-41338-y PMC10491676

[52] Hou R, et al. In vivo manufacture and manipulation of CAR-T cells for better druggability. Cancer Metastasis Rev. 2024;43(3):1075–93. 38592427 10.1007/s10555-024-10185-8

[53] Mhaidly R, Verhoeyen E. Humanized mice are precious tools for preclinical evaluation of CAR T and CAR NK cell therapies. Cancers. 2020;12(7):1915. 32679920 10.3390/cancers12071915 PMC7409195

[54] Lu T, et al. Deep learning-based prediction of the T cell receptor–antigen binding specificity. Nat Mach Intell. 2021;3(10):864–75. 36003885 10.1038/s42256-021-00383-2 PMC9396750

[55] Zeng J, et al. Leveraging artificial intelligence for neoantigen prediction. Cancer Res. 2025;85(13):2376–87. 40101113 10.1158/0008-5472.CAN-24-2553

[56] Xie T, et al. Artificial intelligence: illuminating the depths of the tumor microenvironment. J Translational Med. 2024;22(1):799. 10.1186/s12967-024-05609-6 PMC11360846 39210368

[57] Chang L, et al. Advancing precision medicine: the transformative role of artificial intelligence in immunogenomics, radiomics, and pathomics for biomarker discovery and immunotherapy optimization. Cancer Biology Med. 2025;22(1):33–47. 10.20892/j.issn.2095-3941.2024.0376 PMC11795263 39749734

[58] Ouyang P, et al. Infiltration Characteristics and Regulatory Mechanisms of CD8 + T Lymphocytes in Solid Tumors: Spatial Distribution, Biological Functions, and Interactions with the Immune Microenvironment. Front Immunol. 2025;16:1661545. 41103436 10.3389/fimmu.2025.1661545 PMC12521937

[59] Dutta S, et al. Targets of immune escape mechanisms in cancer: basis for development and evolution of cancer immune checkpoint inhibitors. Biology. 2023;12(2):218. 36829496 10.3390/biology12020218 PMC9952779

[60] Seliger B, Massa C. Immune therapy resistance and immune escape of tumors. Cancers. 2021;13(3):551. 33535559 10.3390/cancers13030551 PMC7867077

[61] Saeed AF. Tumor-Associated Macrophages: Polarization, Immunoregulation, and Immunotherapy. Cells. 2025;14(10):741. 40422244 10.3390/cells14100741 PMC12110377

[62] Chaux A. *Molecular Mechanisms of Targeted Therapy Resistance in Genitourinary Tumors: A Path to New Therapeutic Horizons.* 2025.

[63] Garg P, et al. Emerging therapeutic strategies to overcome drug resistance in cancer cells. Cancers. 2024;16(13):2478. 39001539 10.3390/cancers16132478 PMC11240358

[64] Strohl WR, Naso M. Bispecific T-cell redirection versus chimeric antigen receptor (CAR)-T cells as approaches to kill cancer cells. Antibodies. 2019;8(3):41. 31544847 10.3390/antib8030041 PMC6784091

[65] Phung SK, Miller JS, Felices M. Bi-specific and tri-specific NK cell engagers: the new avenue of targeted NK cell immunotherapy. Mol Diagn Ther. 2021;25(5):577–92. 34327614 10.1007/s40291-021-00550-6

[66] Jørgensen LV, Christensen EB, Barnkob MB, Barington T. The clinical landscape of CAR NK cells. Experimental Hematol Oncol. 2025;14(1):46. 10.1186/s40164-025-00633-8 PMC11951618 40149002

[67] Yi M, et al. Combination strategies with PD-1/PD-L1 blockade: current advances and future directions. Mol Cancer. 2022;21(1):28. 35062949 10.1186/s12943-021-01489-2 PMC8780712

[68] Ahmed S, Mazhar MS, Shabbir MF. Neoantigen-based cancer vaccines: Current innovations, challenges and future directions in personalized immunotherapy. Cancer Immunol Connect. 2024;1(1):1–10.

[69] Aggeletopoulou I, Pantzios S, Triantos C. Personalized Immunity: Neoantigen-Based Vaccines Revolutionizing Hepatocellular Carcinoma Treatment. Cancers. 2025;17(3):376. 39941745 10.3390/cancers17030376 PMC11815775

[70] Naffaa MM, Al-Ewaidat OA, Gogia S, Begiashvili V. Neoantigen-based immunotherapy: advancing precision medicine in cancer and glioblastoma treatment through discovery and innovation. Explor Target Anti-tumor Therapy. 2025;6:1002313. 10.37349/etat.2025.1002313 PMC12040680 40309350

[71] Ghasemi K. Tiragolumab and TIGIT: pioneering the next era of cancer immunotherapy. Front Pharmacol. 2025;16:1568664. 40567374 10.3389/fphar.2025.1568664 PMC12187662

[72] Zaheer S, Sureka N. Unlocking New Frontiers: Novel Immune Targets for Next-Gen Cancer Immunotherapy. Authorea Preprints; 2024. 10.14216/kjco.24322 PMC12415433 40916400

[73] Lu C, Tan Y. Promising immunotherapy targets: TIM3, LAG3, and TIGIT joined the party. Mol Therapy Oncol. 2024;32:1. 10.1016/j.omton.2024.200773 PMC10905042 38596295

[74] Andrews LP, et al. LAG-3 and PD-1 synergize on CD8 + T cells to drive T cell exhaustion and hinder autocrine IFN-γ-dependent anti-tumor immunity. Cell. 2024;187(16):4355–72. e22. 39121848 10.1016/j.cell.2024.07.016 PMC11323044

[75] Qiu X et al. *PD-1 and LAG-3 dual blockade: emerging mechanisms and potential therapeutic prospects in cancer*. 2024, China Anti-Cancer Association. pp. 970–976. 10.20892/j.issn.2095-3941.2024.0436 PMC11667783 39641454

[76] Hofmann M, Thimme R, Schamel WW. PD-1 and LAG-3: synergistic fostering of T cell exhaustion. Signal Transduct Target Therapy. 2024;9(1):291. 10.1038/s41392-024-02000-1 PMC11489778 39424778

[77] Bruss C, et al. Immune checkpoint profiling in humanized breast cancer mice revealed cell-specific LAG-3/PD-1/TIM-3 co-expression and elevated PD-1/TIM-3 secretion. Cancers. 2023;15(9):2615. 37174080 10.3390/cancers15092615 PMC10177290

[78] Pourmir I. Membrane and soluble forms of the immune checkpoint TIM-3 in clear cell renal cell carcinoma: a promising biomarker for cancer immunotherapy. Université Paris Cité; 2024.

[79] Lin A, et al. Natural Killer Cell Immune Checkpoints and Their Therapeutic Targeting in Cancer Treatment. Research. 2025;8:0723. 40463500 10.34133/research.0723 PMC12131497

[80] Croft M. Control of immunity by the TNFR-related molecule OX40 (CD134). Annu Rev Immunol. 2010;28(1):57–78. 20307208 10.1146/annurev-immunol-030409-101243 PMC2882161

[81] Ma Y, et al. Combination of PD-1 inhibitor and OX40 agonist induces tumor rejection and immune memory in mouse models of pancreatic cancer. Gastroenterology. 2020;159(1):306–19. e12. 32179091 10.1053/j.gastro.2020.03.018 PMC7387152

[82] Yadav R, Redmond WL. Current clinical trial landscape of OX40 agonists. Curr Oncol Rep. 2022;24(7):951–60. 35352295 10.1007/s11912-022-01265-5

[83] Sturgill ER, Redmond WL. TNFR agonists: a review of current biologics targeting OX40, 4-1BB, CD27, and GITR. Am J Hematol Oncol. 2017;13(11):4–15.

[84] Chen S, et al. Combination of 4-1BB agonist and PD-1 antagonist promotes antitumor effector/memory CD8 T cells in a poorly immunogenic tumor model. Cancer Immunol Res. 2015;3(2):149–60. 25387892 10.1158/2326-6066.CIR-14-0118

[85] Tian J, Zhang B, Rui K, Wang S. The role of GITR/GITRL interaction in autoimmune diseases. Front Immunol. 2020;11:588682. 33163004 10.3389/fimmu.2020.588682 PMC7581784

[86] Papadakos SP, et al. Exploring the Role of GITR/GITRL Signaling: From Liver Disease to Hepatocellular Carcinoma. Cancers. 2024;16(14):2609. 39061246 10.3390/cancers16142609 PMC11275207

[87] Balmanoukian AS, et al. Safety and clinical activity of MEDI1873, a novel GITR agonist, in advanced solid tumors. Clin Cancer Res. 2020;26(23):6196–203. 32887725 10.1158/1078-0432.CCR-20-0452

[88] Beatty GL, Li Y, Long KB. Cancer immunotherapy: activating innate and adaptive immunity through CD40 agonists. Expert Rev Anticancer Ther. 2017;17(2):175–86. 27927088 10.1080/14737140.2017.1270208 PMC5533512

[89] de Silva S, et al. CD40 enhances type I interferon responses downstream of CD47 blockade, bridging innate and adaptive immunity. Cancer Immunol Res. 2020;8(2):230–45. 31852716 10.1158/2326-6066.CIR-19-0493

[90] Palmer N, Khakoo SI, Sanchez-Elsner T, Vallejo AF. Enhancing natural killer cell anti-tumour activity through macrophage manipulation. Front Immunol. 2025;16:1656925. 40948757 10.3389/fimmu.2025.1656925 PMC12426019

[91] Laskowski TJ, Biederstädt A, Rezvani K. Natural killer cells in antitumour adoptive cell immunotherapy. Nat Rev Cancer. 2022;22(10):557–75. 35879429 10.1038/s41568-022-00491-0 PMC9309992

[92] Huan T, et al. Principles and current clinical landscape of NK cell engaging bispecific antibody against cancer. Hum Vaccines Immunotherapeutics. 2023;19(2):2256904. 10.1080/21645515.2023.2256904 PMC10543353 37772505

[93] Larionova I, Kazakova E, Gerashchenko T, Kzhyshkowska J. New angiogenic regulators produced by TAMs: perspective for targeting tumor angiogenesis. Cancers. 2021;13(13):3253. 34209679 10.3390/cancers13133253 PMC8268686

[94] Huang R, Kang T, Chen S. The role of tumor-associated macrophages in tumor immune evasion. J Cancer Res Clin Oncol. 2024;150(5):238. 38713256 10.1007/s00432-024-05777-4 PMC11076352

[95] Xu J, et al. Dual roles and therapeutic targeting of tumor-associated macrophages in tumor microenvironments. Signal Transduct Target Therapy. 2025;10(1):268. 10.1038/s41392-025-02325-5 PMC12375796 40850976

[96] Aizaz M, et al. The cross-talk between macrophages and tumor cells as a target for cancer treatment. Front Oncol. 2023;13:1259034. 38033495 10.3389/fonc.2023.1259034 PMC10682792

[97] Wang K, et al. The mechanism of action and therapeutic potential of tumor-associated macrophages in tumor immune evasion. Front Immunol. 2025;16:1545928. 40330472 10.3389/fimmu.2025.1545928 PMC12052954

[98] Wang Z, Wu X. Study and analysis of antitumor resistance mechanism of PD1/PD-L1 immune checkpoint blocker. Cancer Med. 2020;9(21):8086–121. 32875727 10.1002/cam4.3410 PMC7643687

[99] Xu-Monette ZY, Zhang M, Li J, Young KH. PD-1/PD-L1 blockade: have we found the key to unleash the antitumor immune response? Front Immunol. 2017;8:1597. 29255458 10.3389/fimmu.2017.01597 PMC5723106

[100] Goodman A, Patel SP, Kurzrock R. PD-1–PD-L1 immune-checkpoint blockade in B-cell lymphomas. Nat reviews Clin Oncol. 2017;14(4):203–20. 10.1038/nrclinonc.2016.168 27805626

[101] Chow A, Perica K, Klebanoff CA, Wolchok JD. Clinical implications of T cell exhaustion for cancer immunotherapy. Nat reviews Clin Oncol. 2022;19(12):775–90. 10.1038/s41571-022-00689-z PMC10984554 36216928

[102] Fang Z, et al. Revolutionizing tumor immunotherapy: unleashing the power of progenitor exhausted T cells. Cancer Biology Med. 2024;21(6):499–512. 10.20892/j.issn.2095-3941.2024.0105 PMC11208905 38825813

[103] Wang N-H, et al. Radiation-induced PD-L1 expression in tumor and its microenvironment facilitates cancer-immune escape: a narrative review. Annals Translational Med. 2022;10(24):1406. 10.21037/atm-22-6049 PMC9843429 36660640

[104] Voronova V, et al. Combination of immune checkpoint inhibitors with radiation therapy in cancer: a hammer breaking the wall of resistance. Front Oncol. 2022;12:1035884. 36544712 10.3389/fonc.2022.1035884 PMC9760959

[105] He R, et al. PD-1 and CTLA-4 inhibitors in combination vs. alone for the treatment of advanced melanoma: A systematic review and meta-analysis. Medicine. 2022;101(41):e30561. 36254050 10.1097/MD.0000000000030561 PMC9575742

[106] Nishio M, et al. Atezolizumab plus chemotherapy for first-line treatment of nonsquamous NSCLC: results from the randomized phase 3 IMpower132 trial. J Thorac Oncol. 2021;16(4):653–64. 33333328 10.1016/j.jtho.2020.11.025

[107] Chen Q-A, et al. Efficacy and Safety of Anti–Programmed Cell Death Protein 1/Programmed Death-Ligand 1 Antibodies Plus Chemotherapy as First-Line Treatment for NSCLC in the People’s Republic of China: a Systematic Review and Meta-Analysis. JTO Clin Res Rep. 2024;5(6):100678. 38846810 10.1016/j.jtocrr.2024.100678 PMC11153918

[108] Powles T, et al. Pembrolizumab plus axitinib versus sunitinib monotherapy as first-line treatment of advanced renal cell carcinoma (KEYNOTE-426): extended follow-up from a randomised, open-label, phase 3 trial. Lancet Oncol. 2020;21(12):1563–73. 33284113 10.1016/S1470-2045(20)30436-8

[109] Plimack ER, et al. Pembrolizumab plus axitinib versus sunitinib as first-line treatment of advanced renal cell carcinoma: 43-month follow-up of the phase 3 KEYNOTE-426 study. Eur Urol. 2023;84(5):449–54. 37500340 10.1016/j.eururo.2023.06.006

[110] Zhao X, Shao C. Radiotherapy-mediated immunomodulation and anti-tumor abscopal effect combining immune checkpoint blockade. Cancers. 2020;12(10):2762. 32992835 10.3390/cancers12102762 PMC7600068

[111] Russell L, Peng KW, Russell SJ, Diaz RM. Oncolytic viruses: priming time for cancer immunotherapy. BioDrugs. 2019;33(5):485. 31321623 10.1007/s40259-019-00367-0 PMC6790338

[112] Wang L, Dunmall LSC, Cheng Z, Wang Y. Remodeling the tumor microenvironment by oncolytic viruses: beyond oncolysis of tumor cells for cancer treatment. J Immunother Cancer. 2022;10(5):e004167. 35640930 10.1136/jitc-2021-004167 PMC9157365

[113] Shalhout SZ, Miller DM, Emerick KS, Kaufman HL. Therapy with oncolytic viruses: progress and challenges. Nat reviews Clin Oncol. 2023;20(3):160–77. 10.1038/s41571-022-00719-w 36631681

[114] Wang J, et al. Metabolic reprogramming of immune cells in the tumor microenvironment. Int J Mol Sci. 2024;25(22):12223. 39596288 10.3390/ijms252212223 PMC11594648

[115] Shi X, et al. Mechanism insights and therapeutic intervention of tumor metastasis: latest developments and perspectives. Signal Transduct Target therapy. 2024;9(1):192. 10.1038/s41392-024-01885-2 PMC11294630 39090094

[116] Jannati S, Patel A, Patnaik R, Banerjee Y. Oleocanthal as a Multifunctional Anti-Cancer Agent: Mechanistic Insights, Advanced Delivery Strategies, and Synergies for Precision Oncology. Int J Mol Sci. 2025;26(12):5521. 40564985 10.3390/ijms26125521 PMC12193433

[117] Papageorgiou A. Biomarkers in Immune Checkpoint Inhibitor Cancer Treatment. Science. 2024;1:100023.

[118] Passaro A, et al. Cancer biomarkers: Emerging trends and clinical implications for personalized treatment. Cell. 2024;187(7):1617–35. 38552610 10.1016/j.cell.2024.02.041 PMC7616034

[119] Jardim DL, Goodman A, de Melo D, Gagliato, Kurzrock R. The challenges of tumor mutational burden as an immunotherapy biomarker. Cancer Cell. 2021;39(2):154–73. 33125859 10.1016/j.ccell.2020.10.001 PMC7878292

[120] Sha D, et al. Tumor mutational burden as a predictive biomarker in solid tumors. Cancer Discov. 2020;10(12):1808–25. 33139244 10.1158/2159-8290.CD-20-0522 PMC7710563

[121] Sun S, et al. The role of neoantigens and tumor mutational burden in cancer immunotherapy: advances, mechanisms, and perspectives. J Hematol Oncol. 2025;18(1):84. 40898324 10.1186/s13045-025-01732-z PMC12406617

[122] Lorenzi M, et al. Epidemiology of microsatellite instability high (MSI-H) and deficient mismatch repair (dMMR) in solid tumors: a structured literature review. J Oncol. 2020;2020(1):1807929.

[123] Ooki A, Osumi H, Yoshino K, Yamaguchi K. Potent therapeutic strategy in gastric cancer with microsatellite instability-high and/or deficient mismatch repair. Gastric Cancer. 2024;27(5):907. 38922524 10.1007/s10120-024-01523-4 PMC11335850

[124] Turabi K, Klute K, Radhakrishnan P. Decoding the dynamics of circulating tumor DNA in liquid biopsies. Cancers. 2024;16(13):2432. 39001494 10.3390/cancers16132432 PMC11240538

[125] Allen TA. The role of circulating tumor cells as a liquid biopsy for cancer: advances, biology, technical challenges, and clinical relevance. Cancers. 2024;16(7):1377. 38611055 10.3390/cancers16071377 PMC11010957

[126] Ahmed J, Das B, Shin S, Chen A. Challenges and future directions in the management of tumor mutational burden-high (TMB-H) advanced solid malignancies. Cancers. 2023;15(24):5841. 38136385 10.3390/cancers15245841 PMC10741991

[127] Ricciuti B, et al. Association of high tumor mutation burden in non–small cell lung cancers with increased immune infiltration and improved clinical outcomes of PD-L1 blockade across PD-L1 expression levels. JAMA Oncol. 2022;8(8):1160–8. 35708671 10.1001/jamaoncol.2022.1981 PMC9204620

[128] Awosika JA, Gulley JL, Pastor DM. Deficient Mismatch Repair and Microsatellite Instability in Solid Tumors. Int J Mol Sci. 2025;26(9):4394. 40362635 10.3390/ijms26094394 PMC12072705

[129] Bhamidipati D, Subbiah V. Tumor-agnostic drug development in dMMR/MSI-H solid tumors. Trends Cancer. 2023;9(10):828–39. 37517955 10.1016/j.trecan.2023.07.002

[130] Ambrosini M, et al. Epidemiology, pathogenesis, biology and evolving management of MSI-H/dMMR cancers. Nat Reviews Clin Oncol. 2025;2:1–23. 10.1038/s41571-025-01015-z 40181086

[131] Al-Oudah GA, Alfalluji WL, Al-Ajrash AM. Checkpoint Inhibitors in Cancer Immunotherapy: Mechanisms of Action and Resistance. J Biomed Biochem. 2025;4(2):124–49.

[132] Kelloff G, Sigman CC. *Opportunities for Liquid Biopsies to Meet the Challenges of Precision Medicine*, in *Circulating Tumor Cells: Advances in Liquid Biopsy Technologies*. Springer; 2023. pp. 443–60.

[133] Schoenfeld AJ, Hellmann MD. Acquired resistance to immune checkpoint inhibitors. Cancer Cell. 2020;37(4):443–55. 32289269 10.1016/j.ccell.2020.03.017 PMC7182070

[134] Mainini F, Eccles MR. Lipid and polymer-based nanoparticle siRNA delivery systems for cancer therapy. Molecules. 2020;25(11):2692. 32532030 10.3390/molecules25112692 PMC7321291

[135] Gu X, Minko T. Targeted nanoparticle-based diagnostic and treatment options for pancreatic cancer. Cancers. 2024;16(8):1589. 38672671 10.3390/cancers16081589 PMC11048786

[136] Youssef E, Fletcher B, Palmer D. Enhancing precision in cancer treatment: the role of gene therapy and immune modulation in oncology. Front Med. 2025;11:1527600. 10.3389/fmed.2024.1527600 PMC11769984 39871848

[137] Bergamaschi C, et al. Innovative strategies for T cell engagers for cancer immunotherapy. Mabs. Taylor & Francis; 2025. 10.1080/19420862.2025.2531223 PMC12258164 40641219

[138] Huang S, Van Duijnhoven SM, Sijts AJ, Van Elsas A. Bispecific antibodies targeting dual tumor-associated antigens in cancer therapy. J Cancer Res Clin Oncol. 2020;146(12):3111–22. 32989604 10.1007/s00432-020-03404-6 PMC7679314

[139] van de Donk NW, Zweegman S. T-cell-engaging bispecific antibodies in cancer. Lancet. 2023;402(10396):142–58. 37271153 10.1016/S0140-6736(23)00521-4

[140] Zhang C, Zhuang Q, Liu J, Liu X. Synthetic biology in chimeric antigen receptor T (CAR T) cell engineering. ACS Synth Biol. 2022;11(1):1–15. 35005887 10.1021/acssynbio.1c00256

[141] Dai H, Wang Y, Lu X, Han W. Chimeric antigen receptors modified T-cells for cancer therapy. J Natl Cancer Inst. 2016;108(7):djv439. 26819347 10.1093/jnci/djv439 PMC4948566

[142] Wells K, Liu T, Zhu L, Yang L. Immunomodulatory nanoparticles activate cytotoxic T cells for enhancement of the effect of cancer immunotherapy. Nanoscale, 2024. 10.1039/d4nr01780c PMC12139611 39257225

[143] Peng L, et al. CAR-T and CAR-NK as cellular cancer immunotherapy for solid tumors. Cell Mol Immunol. 2024;21(10):1089–108. 39134804 10.1038/s41423-024-01207-0 PMC11442786

[144] Rafii S, et al. Advancing CAR T-Cell Therapy in Solid Tumors: Current Landscape and Future Directions. Cancers. 2025;17(17):2898. 40940995 10.3390/cancers17172898 PMC12428560

[145] Selmi I. Oncolytic Viruses: Mechanisms, Engineering Strategies, and Clinical Advances. Adv Ther J. 2025;7(24):32–41.

[146] Jin K-T, et al. Oncolytic virotherapy in solid tumors: the challenges and achievements. Cancers. 2021;13(4):588. 33546172 10.3390/cancers13040588 PMC7913179

[147] Bayode MT, et al. Modulating the immunosuppressive tumor microenvironment: Multi-synergistic cancer therapeutic approach with oncolytic viruses. World J Adv Res Reviews. 2024;21:543–59.

[148] Jenkins FG, Johnson JE, Collichio F, Ollila DW. Talimogene laherparepvec and novel injectable oncolytic viruses in the management of metastatic melanoma. J Transl Genet Genom. 2021;5:396–413.

[149] Wang Z, Gu Y, Sun X, Huang H. Computation strategies and clinical applications in neoantigen discovery towards precision cancer immunotherapy. Biomark Res. 2025;13(1):96. 40629481 10.1186/s40364-025-00808-9 PMC12239460

[150] Gupta RG, Li F, Roszik J, Lizée G. Exploiting tumor neoantigens to target cancer evolution: current challenges and promising therapeutic approaches. Cancer Discov. 2021;11(5):1024–39. 33722796 10.1158/2159-8290.CD-20-1575 PMC8102318

[151] Bradley P. Structure-based prediction of T cell receptor: peptide-MHC interactions. elife. 2023;12:e82813. 36661395 10.7554/eLife.82813 PMC9859041

[152] Li X, et al. Neoantigen cancer vaccines: a new star on the horizon. Cancer Biology Med. 2024;21(4):274–311. 10.20892/j.issn.2095-3941.2023.0395 PMC11033713 38164734

[153] Dunn GP, Sherpa N, Manyanga J, Johanns TM. Considerations for personalized neoantigen vaccination in Malignant glioma. Adv Drug Deliv Rev. 2022;186:114312. 35487282 10.1016/j.addr.2022.114312

[154] Su S, et al. Recent advances in neoantigen vaccines for treating non-small cell lung cancer. Thorac Cancer. 2023;14(34):3361–8. 37905603 10.1111/1759-7714.15126 PMC10693939

[155] Yuan Z, et al. Extracellular matrix remodeling in tumor progression and immune escape: from mechanisms to treatments. Mol Cancer. 2023;22(1):48. 36906534 10.1186/s12943-023-01744-8 PMC10007858

[156] Yang Y, et al. Tumor-associated macrophages remodel the suppressive tumor immune microenvironment and targeted therapy for immunotherapy. J Experimental Clin Cancer Res. 2025;44(1):145. 10.1186/s13046-025-03377-9 PMC12083052 40380196

[157] Wang H, et al. Shaping the immune-suppressive microenvironment on tumor‐associated myeloid cells through tumor‐derived exosomes. Int J Cancer. 2024;154(12):2031–42. 38500385 10.1002/ijc.34921

[158] Fu Z, et al. Tumour hypoxia-mediated immunosuppression: mechanisms and therapeutic approaches to improve cancer immunotherapy. Cells. 2021;10(5):1006. 33923305 10.3390/cells10051006 PMC8146304

[159] Gouarderes S, Mingotaud A-F, Vicendo P, Gibot L. Vascular and extracellular matrix remodeling by physical approaches to improve drug delivery at the tumor site. Expert Opin Drug Deliv. 2020;17(12):1703–26. 32838565 10.1080/17425247.2020.1814735

[160] Arner EN, Rathmell JC. Metabolic programming and immune suppression in the tumor microenvironment. Cancer Cell. 2023;41(3):421–33. 36801000 10.1016/j.ccell.2023.01.009 PMC10023409

[161] Bilotta MT, Antignani A, Fitzgerald DJ. Managing the TME to improve the efficacy of cancer therapy. Front Immunol. 2022;13:954992. 36341428 10.3389/fimmu.2022.954992 PMC9630343

[162] Liu S, et al. Drug-induced tolerant persisters in tumor: Mechanism, vulnerability and perspective implication for clinical treatment. Mol Cancer. 2025;24(1):150. 40413503 10.1186/s12943-025-02323-9 PMC12102949

[163] Lv D, et al. Crosstalk between T lymphocyte and extracellular matrix in tumor microenvironment. Front Immunol. 2024;15:1340702. 38690275 10.3389/fimmu.2024.1340702 PMC11058664

[164] Ju Y, et al. Nanostructured particles assembled from natural building blocks for advanced therapies. Chem Soc Rev. 2022;51(11):4287–336. 35471996 10.1039/d1cs00343g

[165] Li D, Rudloff U. Emerging therapeutics targeting tumor-associated macrophages for the treatment of solid organ cancers. Expert Opin Emerg Drugs. 2025;30(2):109–47. 40353504 10.1080/14728214.2025.2504376 PMC12232465

[166] Haist M, Stege H, Grabbe S, Bros M. The functional crosstalk between myeloid-derived suppressor cells and regulatory T cells within the immunosuppressive tumor microenvironment. Cancers. 2021;13(2):210. 33430105 10.3390/cancers13020210 PMC7827203

[167] Bullock K, Richmond A. Suppressing MDSC recruitment to the tumor microenvironment by antagonizing CXCR2 to enhance the efficacy of immunotherapy. Cancers. 2021;13(24):6293. 34944914 10.3390/cancers13246293 PMC8699249

[168] Newsome RC, et al. Interaction of bacterial genera associated with therapeutic response to immune checkpoint PD-1 blockade in a United States cohort. Genome Med. 2022;14(1):35. 35346337 10.1186/s13073-022-01037-7 PMC8961902

[169] Peng Z, et al. The gut microbiome is associated with clinical response to anti–PD-1/PD-L1 immunotherapy in gastrointestinal cancer. Cancer Immunol Res. 2020;8(10):1251–61. 32855157 10.1158/2326-6066.CIR-19-1014

[170] Zhu C, et al. Gut microbiota and metabolites signatures of clinical response in anti-PD-1/PD-L1 based immunotherapy of biliary tract cancer. Biomark Res. 2024;12(1):56. 38831368 10.1186/s40364-024-00607-8 PMC11149318

[171] Fertitta V, et al. Akkermansia muciniphila-and Pathogenic Bacteria-Derived Endotoxins Differently Regulate Human Dendritic Cell Generation and γδ T Lymphocyte Activation. Biomolecules. 2024;14(12):1571. 39766278 10.3390/biom14121571 PMC11673428

[172] Han EJ, Ahn J-S, Chae YJ, Chung H-J. Immunomodulatory Roles of Faecalibacterium prausnitzii and Akkermansia muciniphila in Autoimmune Diseases: Mechanistic Insights and Therapeutic Potential. Clin Rev Allergy Immunol. 2025;68(1):77. 40759811 10.1007/s12016-025-09093-8

[173] Tran D. Impact of concurrent antibiotic & CORTICOSTEROID use on Efficacy of immune checkpoint inhibitors. University of Minnesota; 2023.

[174] Elkrief A, et al. Gut Microbiota in Immuno-Oncology: A Practical Guide for Medical Oncologists with a Focus on Antibiotics Stewardship. Am Soc Clin Oncol Educational Book. 2025;45(3):e472902. 40262063 10.1200/EDBK-25-472902

[175] Siddiqui MA, Usmani A, Ansari MN, Almoselhy RI. Immune-related adverse events in immunotherapy: Challenges in diagnosis, monitoring, and management. Toxicol Rep. 2025;1:102036. 10.1016/j.toxrep.2025.102036 PMC12433800 40959381

[176] Sainatham C, et al. The current socioeconomic and regulatory landscape of immune effector cell therapies. Front Med. 2024;11:1462307. 10.3389/fmed.2024.1462307 PMC11652178 39697210

[177] Fountzilas E, et al. Convergence of evolving artificial intelligence and machine learning techniques in precision oncology. NPJ Digit Med. 2025;8(1):75. 39890986 10.1038/s41746-025-01471-y PMC11785769

[178] Suura SR. Integrating Artificial Intelligence, Machine Learning, and Big Data with Genetic Testing and Genomic Medicine to Enable Earlier, Personalized Health Interventions. Deep Science Publishing; 2025.

[179] Elfatimi E, Lekbach Y, Prakash S, BenMohamed L. Artificial intelligence and machine learning in the development of vaccines and immunotherapeutics—yesterday, today, and tomorrow. Front Artif Intell. 2025;8:1620572. 40756816 10.3389/frai.2025.1620572 PMC12313644

[180] Kong H. Advances in personalized Cancer vaccine development: AI applications from neoantigen discovery to mRNA formulation. BioChem. 2025;5(2):5.

[181] Omar M et al. *Multiplex imaging analysis in pathology: a comprehensive review on analytical approaches and digital toolkits.* arXiv preprint arXiv:2411.00948, 2024.

[182] Bungaro C, Guida M, Apollonio B. Spatial proteomics of the tumor microenvironment in melanoma: current insights and future directions. Front Immunol. 2025;16:1568456. 40443654 10.3389/fimmu.2025.1568456 PMC12119572

[183] Alamri AM, Assiri AA, Khan B, Khan NU. Next-generation oncology: integrative therapeutic frontiers at the crossroads of precision genomics, immuno-engineering, and tumor microenvironment modulation. Med Oncol. 2025;42(11):1–20. 10.1007/s12032-025-03042-3 40974488

[184] Olawade DB, et al. Integrating AI into cancer immunotherapy—A narrative review of current applications and future directions. Diseases. 2025;13(1):24. 39851488 10.3390/diseases13010024 PMC11764268

[185] Ekundayo F. Reinforcement learning in treatment pathway optimization: A case study in oncology. Int J Sci Res Archive. 2024;13(02):2187–205.

[186] Abdo L, Batista-Silva LR, Bonamino MH. Cost-effective strategies for CAR-T cell therapy manufacturing. Mol Therapy Oncol. 2025;33:2. 10.1016/j.omton.2025.200980 PMC12022644 40291594

[187] Li Y-R, Zhu Y, Halladay T, Yang L. In vivo CAR engineering for immunotherapy. Nat Rev Immunol. 2025;2:1–20. 10.1038/s41577-025-01174-1 40379910

[188] Esmaeilzadeh A, et al. State of the art in CAR-based therapy: In vivo CAR production as a revolution in cell-based cancer treatment. Cell Oncol. 2025;2:1–25. 10.1007/s13402-025-01056-7 PMC12238164 40261561

[189] Tretbar US, et al. Non-viral vectors for chimeric antigen receptor immunotherapy. Nat Reviews Methods Primers. 2024;4(1):74.

[190] Moretti A, et al. The past, present, and future of non-viral CAR T cells. Front Immunol. 2022;13:867013. 35757746 10.3389/fimmu.2022.867013 PMC9218214

